# Clinical progression and genetic pathways in body-first and brain-first Parkinson’s disease

**DOI:** 10.1186/s13024-025-00866-5

**Published:** 2025-06-20

**Authors:** Massimiliano Passaretti, Daniel Veréb, Mite Mijalkov, Yu-Wei Chang, Hang Zhao, Blanca Zufiria-Gerbolés, Jiawei Sun, Giovanni Volpe, Natalia Rivera, Matteo Bologna, Joana B. Pereira

**Affiliations:** 1 Department of Clinical Neuroscience, Karolinska Institutet, K8 Klinisk neurovetenskap, K8 Neuro Pereira, 171 77, Stockholm, Sweden; 2 Department of Human Neurosciences, Sapienza University of Rome, Rome, Italy; 3 Department of Physics, University of Gothenburg, Gothenburg, Sweden; 4 Immunology and Respiratory Medicine Division, Department of Medicine Solna, Karolinska Institutet, Center for Molecular Medicine, Karolinska University Hospital, Karolinska University Hospital, Solna, Stockholm, Sweden; 5 Rheumatology Division, Department of Medicine Solna, Karolinska Institutet, Center for Molecular Medicine, Karolinska University Hospital, Karolinska University Hospital, Solna, Stockholm, Sweden; 6 IRCCS Neuromed, Pozzilli, Italy

**Keywords:** Parkinson’s disease, Body-first, Brain-first, Progression pathways

## Abstract

**Background:**

Parkinson’s disease (PD) is a highly heterogeneous disorder with distinct phenotypes that can develop well before motor symptoms appear. Recently, two main phenotypes based on the different pathological spreading patterns of PD have been proposed: “body-first”, where α-synuclein pathology begins in the peripheral nervous system and spreads symmetrically from bottom-up, and “brain-first”, where pathology starts in the brain and spreads asymmetrically downwards. However, no studies have assessed these phenotypes across both prodromal and clinical PD stages, tracked their pathological progression in vivo or identified potential underlying biological mechanisms.

**Methods:**

To address this, we analyzed 910 prodromal and 1120 clinical PD cases with comprehensive longitudinal clinical, imaging, and genetic data from the Parkinson Progression Marker Initiative over a 12-year period.

**Results:**

Our findings revealed that both prodromal and clinical groups with body-first symptoms exhibited greater motor dysfunction, anxiety, and depression at baseline; as well as worse longitudinal motor progression and attention decline compared to brain-first cases. The body-first and brain-first phenotypes were stable over time and predicted conversion to clinical PD in prodromal cases, and were also found using unsupervised deep learning analyses. Additionally, body-first cases displayed more pronounced changes in the caudal LC, as well as symmetrical alterations in the striatum and glymphatic system, consistent with the traditional bottom-up progression described by Braak’s staging of α-synuclein pathology and the more symmetric distribution proposed for body-first PD. In contrast, brain-first cases exhibited changes in the rostral LC and asymmetric alterations in the striatum and glymphatic system, suggesting a top-down progression. Genetic analysis also identified new specific single nucleotide polymorphisms associated with PD phenotypes, such as *TRIM40* and *IP6K2*, linked to worse motor and cognitive outcomes in prodromal cases.

**Conclusions:**

These findings emphasize the importance of recognizing body-first and brain-first PD as distinct entities with unique clinical, imaging, and genetic profiles, paving the way for targeted and personalized therapeutic strategies that address the specific pathophysiological mechanisms of PD.

**Supplementary Information:**

The online version contains supplementary material available at 10.1186/s13024-025-00866-5.

## Introduction

The clinical diagnosis of Parkinson’s disease (PD) is preceded by pathological processes that begin long before the appearance of motor symptoms [1, 2]. The definition of disease biomarkers during this prodromal phase is therefore important to identify individuals in the very early PD stages [3]. However, increasing evidence indicates that PD, in both its prodromal and clinical stages, is not uniform but consists of distinct phenotypes with specific clinical features, pathogenesis, and progression patterns, suggesting that different biological mechanisms may underlie this heterogeneity [1, 4, 5]. 

In particular, the Synuclein Origin and Connectome (SOC) model suggests that the initial aggregation and spread of α-synuclein, the neuropathological hallmark of PD, may occur either in the brain or in the body, resulting in two main pathophysiological phenotypes [6]. In the “body-first” phenotype, α-synuclein aggregation starts in the peripheral nervous system, and spreads more symmetrically to the brainstem, affecting the loci coerulei (LCs), striatal dopaminergic uptake on both brain hemispheres and neocortex bilaterally in a bottom-up fashion. This leads to early autonomic dysfunction and sleep disturbances, followed by more symmetric motor impairment with axial motor symptoms. In contrast, the “brain-first” phenotype is characterized by initial α-synuclein aggregation in the rostral parts of the central nervous system, where unilateral connections dominate. The disease then descends more asymmetrically in a top-down manner, resulting in early, asymmetric motor symptoms with slower disease progression and fewer non-motor disturbances. Thus, while the body-first phenotype follows the classical Braak’s staging of PD accompanied by a symmetrical distribution according to the SOC model, with pathology possibly remaining undetected in the early disease stages, the brain-first phenotype exhibits a more complex pattern.

While there is growing neuropathological and animal model support for the SOC model [7–11]its results have not yet been tested longitudinally in both prodromal and clinically diagnosed body-first and brain-first PD. If this hypothesis is true, each phenotype should have specific biomarkers detectable from the prodromal stages and be associated with a specific spreading pattern of pathology in vivo as well as biological and genetic alterations that determine their distinct clinical characteristics. Furthermore, it is currently unknown how these phenotypes progress over time, their conversion rates from prodromal to clinical PD, and how stable and comparable they are against existing motor and non-motor PD classifications. These important yet unresolved questions have hindered the widespread adoption of the body-first and brain-first PD classification, which could greatly improve early diagnosis and disease monitoring in research and clinical practice.

Our study aims to address these questions by using comprehensive multimodal data from a well-characterized longitudinal study of 12 years [12]. We will attempt to verify whether the body-first and brain-first phenotypes observed in clinically diagnosed PD can also be identified in the prodromal stages using accessible and widely available clinical tests. This will involve evaluating motor and non-motor clinical trajectories across both clinical and prodromal stages of the disease. The recognition of neurodegenerative disease phenotypes in the prodromal stage, while presenting multiple challenges, is essential for improving diagnostic precision and optimising the timing and targeting of neuroprotective strategies to enhance their therapeutic impact [13]. Additionally, we will use different imaging measures reflecting noradrenergic, dopaminergic, and glymphatic system dysfunction to assess alterations across different Braak’s stages and establish whether the two phenotypes exhibit a top-down or bottom-up pattern of neurodegeneration. Finally, we will uncover genes that predispose to the development of one phenotype and determine its clinical progression. Our unique combination of clinical, neuroimaging, and genetic measures puts us in the ideal position to establish the prognostic value of these pathophysiological phenotypes and identify new imaging and genetic markers that characterize them, enhancing the application of individualized medicine approaches in PD.

## Methods

### Study participants

We analysed data from 1120 clinically diagnosed and 910 prodromal Parkinson’s disease (PD) individuals enrolled in the Parkinson Progression Marker Initiative (PPMI) as of 31 May 2023, a study focused on identifying PD biomarkers and causes [12]. Clinically diagnosed PD patients met standard diagnostic criteria, were in Hoehn and Yahr stages I or II, and showed dopamine transporter deficits on DaTSCAN imaging. For the purposes of this study, we excluded patients who were diagnosed with a non-PD neurodegenerative disorder during the study follow-up such as Multisystem Atrophy (*n* = 14), Dementia with Lewy Bodies (*n* = 8), Corticobasal Dementia (*n* = 2) and Progressive Supranuclear Palsy (*n* = 3). We did not include individuals with Scans Without Evidence of Dopaminergic Deficit (SWEDD) to avoid the introduction of potential biases [14]. Prodromal cases exhibited hyposmia, REM sleep behaviour disorder (RBD), or had single pathogenic variants in Glucosylceramidase Beta (*GBA*), Leucine-Rich Repeat Kinase 2 (*LRRK2*), or Synuclein Alpha (*SNCA*) genes, all of which are associated with a high risk of progression to PD [15]. 

All participants underwent comprehensive clinical assessments at baseline and over a 12-year follow-up period. These assessments included evaluations of daily living activities (UPDRS I and II), motor function (UPDRS III), and motor complications due to therapy (UPDRS IV) using the unified Parkinson’s disease rating scale (UPDRS) [16]. Neuropsychological assessments included global cognition (Montreal Cognitive Assessment, MoCA), visuospatial abilities (Benton’s judgment of line orientation test), memory (Hopkins verbal learning test-revised, HVLT‐R), executive functions (semantic fluency; letter and number sequencing test, LNS), attention (symbol digit modalities test, SDMT), depression (Geriatric Depression Scale, GDS), and anxiety (State-Trait Anxiety Inventory, STAI) [17]. 

Participants were classified into body-first and brain-first phenotypes based on the presence at baseline assessment of RBD and autonomic symptoms using the RBD Questionnaire (RBDSQ) [18] and the Scale for Outcomes in Parkinson’s Disease - Autonomic (SCOPA-AUT) [19]respectively. Participants with a score ≥ 1 standard deviation above the mean of a reference group of 263 healthy controls (Table S1) on either the RBDSQ, the SCOPA-AUT, or both were classified as body-first. Those with scores within the normal range for both scales were classified as brain-first. Additionally, we compared this classification with other classical clinical systems, including tremor-dominant versus non-tremor dominant PD based on the presence or absence of resting tremor [20, 21] and cognitively normal versus PD with mild cognitive impairment (MCI) based on the PD-MCI guidelines of the Movement Disorder Society (MDS) Task Force [22, 23]. 

The PPMI study was approved by the institutional review boards of all participating sites, and informed consent was obtained from all participants before inclusion in the study.

### Imaging analyses

To examine the progression of pathology from bottom-up or top-down brain structures in body-first and brain-first PD, we utilized three imaging modalities: functional magnetic resonance imaging (fMRI), dopaminergic imaging (DaTSCAN), and diffusion-weighted imaging (DWI). According to Braak’s staging system of α-synuclein pathology, early stages (I-II) are characterized by changes in the LC, a critical brain region involved in autonomic functions and sleep, which are typically impaired in body-first PD [6, 24]. The following stages (III-IV) involve α-synuclein aggregates in the striatum, commonly used to assess dopaminergic denervation, which is expected to be more asymmetric in brain-first PD [25]. Finally, the advanced stages (V-VI) are marked by pathological changes in the neocortex, assessable through the glymphatic system, whose activity is linked to sleep waves and may be implicated in body-first PD [26, 27]. Detailed descriptions of the imaging sequences and analyses for each brain region according to Braak’s staging are provided below.

#### Functional magnetic resonance imaging

To explore propagation patterns in body-first and brain-first PD, we applied a gradient-based functional connectivity analysis to the LC using a pipeline previously described [28]. Resting-state fMRI was conducted on 204 clinical and 202 prodromal PD individuals using a 3 Tesla scanner with the following parameters: echo time = 30 ms, repetition time = 2500 ms, voxel size = 3.3 × 3.3 × 3.3 mm³, matrix size = 64 × 64, field of view = 224 × 224 mm², and flip angle = 80°, with a scan duration of 10 min. T1-weighted scans were also acquired with the following parameters: echo time = 2–6 s, repetition time = 5–11 s, voxel size = 1 × 1 × 1–1.2 mm³, matrix size = 256 × 256, field of view = 256 mm, and flip angle = 9°.

Preprocessing of fMRI scans was performed using the FMRIB Software Library (FSL) [29]. The first five volumes were excluded to achieve steady-state magnetization, and the remaining volumes were corrected for motion using Motion Correction FMRIB’s Linear Image Registration Tool (MCFLIRT) [30]. Non-brain structures were removed with Brain Extraction Tool (BET) [31]and the images were then slice-time corrected, globally normalized, and smoothed with a 3 mm Gaussian kernel constrained to the LC to avoid signal leakage from adjacent brainstem structures or the fourth ventricle. Motion artifacts were addressed using Independent Component Analysis-based Automatic Removal of Motion Artifact (ICA-AROMA) with default non-aggressive cleanup [32, 33]and signals from white matter and cerebrospinal fluid were regressed out. A high-pass temporal filter with a 0.01 Hz cutoff was applied, and images were registered to the standard 2 mm Montreal Neurological Institute 152 (MNI152) template using a two-stage boundary-based registration algorithm.

The LC mask was defined using a recent consensus derived from neuromelanin-sensitive MRI sequences [34]transformed to standard MNI152 space, and downsampled to match the fMRI dataset resolution. LC gradients were extracted using a connectopic mapping approach with the ConnGrads toolbox [35]which involved deriving functional connectivity matrices between LC voxels and cortical brain regions (defined by the Glasser-atlas) [36]. Similarity matrices were constructed with the eta-squared measure and decomposed using the Laplacian eigenmaps algorithm [37] to identify functional gradients, summarized with a trend surface modelling approach [38]. Bayesian linear regression was employed to fit a third-order trend surface model, incorporating coefficients for spatial axes (x, y, z) and their second and third powers [39–41]. These coefficients allowed us to assess whether the rostral (superior) or caudal (inferior) parts of the LC exhibited higher or lower connectivity, indicating bottom-up or top-down disease progression patterns.

#### Dopaminergic imaging

The striatum is a key region in PD, where dopaminergic deficits typically appear in Braak stages III-IV, coinciding with symptom onset and diagnosis. To evaluate dopaminergic deficits, 1120 PD patients underwent dopamine transporter imaging (DaTSCAN) using Single Photon Emission Computed Tomography (SPECT). Imaging preprocessing involved spatial normalization, reconstruction, and attenuation correction to standardize DaTSCAN images [42]. Dopamine transporter binding was quantified by calculating count densities from the caudate and putamen, with the occipital cortex serving as a reference region. The specific binding ratio (SBR) was determined using the formula: SBR = (striatal region count density / occipital count density) − 1. Dopaminergic binding was assessed separately in the more and less affected brain hemispheres, contralateral to the side with more severe motor symptoms since we expected higher asymmetry in brain-first PD [6]. DaTSCANs were not available for prodromal cases, as they are typically performed only in symptomatic individuals.

#### Diffusion weighted imaging

Alterations in the glymphatic system, which may affect the clearance of toxic proteins like α-synuclein, are linked to neocortical impairment in Braak stages V-VI [43]. We assessed glymphatic system activity in 174 clinical and 68 prodromal PD cases using diffusion-weighted imaging (DWI) with the following parameters: repetition time = 900 ms, echo time = 88 ms, voxel size = 2 × 2 × 2 mm³, flip angle = 90°, 72 slices, 64 gradient directions, b-values of 1000 s/mm² and a non-diffusion weighed reference volume. Preprocessing was performed using FSL, including eddy-current correction, head motion correction, and skull stripping. Five subjects were excluded due to motion-related artifacts.

To quantify glymphatic activity in perivascular spaces (DTI-ALPS), we followed a method described by Taoka et al. [44]. This involved fitting diffusion metric images and generating color-coded fractional anisotropy (colFA) and diffusivity maps (Dx, Dy, Dz) along the x-, y-, and z-axes. Spherical regions of interest (ROI) were placed on colFA maps at the level of the lateral ventricle body, and mean diffusivity values from Dx, Dy, and Dz maps were extracted from these ROIs. The DTI-ALPS index was calculated using specific diffusivity measures: x-axis diffusivities in projection fibers (Dx_proj_) and association fibers (Dx_assoc_), as well as y-axis diffusivity in projection fibers (Dy_proj_) and z-axis diffusivity in association fibers (Dz_assoc_). A higher value of DTI-ALPS index indicates better levels of glymphatic system function, while a lower value suggests impaired function.

### Genetic analyses

To investigate the genetic basis of distinct PD phenotypes, we analysed single nucleotide polymorphisms (SNPs) using whole-blood DNA specimens, following established protocols [45, 46]. No individuals with a recognized genetic mutation for PD was included in the genetic association analysis of SNPs with clinical PD phenotypes. Genotypes associated with PD risk were derived from a large case-control study and a genome-wide association (GWAS) meta-analysis protocol, with all available SNPs being considered in the analyses [45, 46]. Quality control measures excluded subjects with more than 5% missing SNPs and SNPs with a call rate below 85%. Markers were tested for Hardy-Weinberg equilibrium (*p* < 0.01), with a less stringent criterion to retain informative SNPs. We focused on the genetic impact of SNPs linked to body-first and brain-first PD on the clinical progression of prodromal cases, examining how specific genetic variants influence disease progression.

### Unsupervised learning analyses

To determine whether the body-first and brain-first phenotypes could be identified without relying on predefined clinical criteria or tests, we used a variational autoencoder (VAE) [47–49]. A VAE is a deep learning model that compresses input data into a low-dimensional latent space using an encoder and then reconstructs the data as accurately as possible using a decoder. This approach helps reveal patterns in the data without making prior assumptions. For our analysis, we used baseline data available for all subjects, including clinical, glymphatic, and genetic data. In total, we included 205 data points, with 164 used for training and 21 for testing. The VAE’s architecture compressed 11 input features into 2 latent features, which were then used to reconstruct the original data. The VAE architecture compressed 11 input features into 2 latent features, which were then used to reconstruct the original data. The encoder network started with an input layer of 11 neurons (representing the features for each subject), followed by a fully connected layer with 16 neurons, a Rectified Linear Unit (ReLU) activation layer, and a fully connected layer with 4 neurons leading to the latent space. The decoder mirrored the encoder, consisting of two fully connected layers and a linear output layer.

The model was trained using the Evidence Lower Bound (ELBO) loss function over 1000 epochs, with a batch size of 40 and a learning rate of 0.001 to fine-tune its performance. The VAE analyses were conducted using MATLAB version 2023b.

### Statistical analyses

Statistical analyses were conducted using R version 4.2.2, adjusting for multiple comparisons with false discovery rate (FDR) corrections (q = 0.05) [50].

#### Clinical variables

Differences in baseline clinical measures between body-first and brain-first PD, as well as between body-first and brain-first prodromal groups, were assessed using the Wilcoxon rank-sum test (Mann-Whitney U) for continuous variables and the chi-squared test for binary variables. To investigate if individual genetic variants, such as mutations in *GBA*, *LRRK2*, *PRKN*, *PINK1* and *SNCA* genes, are associated with PD phenotypes, we performed logistic regression analyses using PD phenotypes as the outcome and genetic groups as predictors. Age, sex, education, and disease duration (in clinical stages) were included as covariates. Longitudinal trajectories of clinical measures were analysed separately using linear mixed-effect models, including fixed effects for group, time, age, sex, disease duration, and education (for cognitive variables), along with interactions between group and time and random intercepts. We conducted sensitivity analyses classifying body-first and brain-first individuals using only autonomic symptoms, to verify that our results were not only driven by the effect of RBD. We also perform a supplementary set of analyses using as covariates H&Y PD status, the presence of cardiovascular risk factors or diseases, and the use of Clonazepam, a drug that could mask RBD symptoms [51]. Cox regression models, adjusted for age, sex, and education, were used to assess the risk of conversion to PD in body-first and brain-first prodromal subjects, with the time of PD diagnosis as the main outcome. To estimate the risk of conversion to PD dementia of body-first and brain-first PD patients, we used a similar Cox regression model with the time to reach a MoCA inferior to 21 as the main outcome [52]. Additionally, we run Cox models accounting for H&Y PD status (in clinical stages), cardiovascular risk factors, and diseases, also excluding the potential confounding effect due to Clonazepam usage. The proportional hazard assumption was verified using Scaled Schoenfeld’s residuals.

We conducted sensitivity analyses to compare the body-first/brain-first classification with other clinical systems, including tremor-dominant vs. non-tremor-dominant and cognitively normal vs. mild cognitive impairment (MCI). Only for this analysis, we applied classification criteria at each time point to calculate wrong reclassification, defined as a shift to a less severe phenotype (i.e., from MCI to cognitively normal), and reclassifications into a more severe phenotype, considered to reflect disease progression (i.e., from cognitively normal to MCI). The percentage of incorrect reclassifications was calculated for the entire baseline population.

#### Imaging variables

For LC connectivity, we performed a back-projection analysis, calculating Pearson’s correlation coefficients between extreme gradient regions and cortical parcels. Functional connections were thresholded at *R* = 0.15, and cortical regions were categorized based on their connectivity with rostral or caudal LC parts. 

To quantify the similarity with the middle portion of the gradient, we applied the following procedure. First, we divided the LC into 5 sections (i = 1–5) along the z-axis (from z = 23 to z = 28), where $\:\text{i}=1$ and $\:\text{i}=2$ correspond to the caudal part of LC (z = 23–24), $\:\text{i}=3$ corresponds to the middle LC section (z = 25–26), and $\:\text{i}=4$ and $\:\text{i}=5$ to the rostral LC (z = 27–28). After this division, we averaged the connectivity gradient of the voxels within each section. We then computed the difference between each Sect. (2 caudal, 2 rostral) with respect to the middle reference ($\:\text{i}=3)$. The similarity with the middle section (SMS_i_) was defined as: $\:\text{S}\text{M}{\text{S}}_{\text{i}}=\:-1\times\:\:\left|{\text{S}}_{\text{i}}-{\text{S}}_{\text{m}\text{i}\text{d}\text{d}\text{l}\text{e}}\right|$, where $\:{\text{S}}_{\text{i}}$ is the average connectivity gradient within each caudal/rostral section and $\:{\text{S}}_{\text{m}\text{i}\text{d}\text{d}\text{l}\text{e}}$ is the average connectivity gradient of the middle section. The resulting values for each section were normalised to a 0–1 scale, where 1 indicates perfect similarity with the middle section and 0 indicates no similarity. A bootstrap-based approach was used to compare functional gradients between groups.

Dopaminergic uptake was analysed using linear mixed models with DaTSCAN striatal values as outcome variables, including group, time, their interaction, and random intercepts, with age, sex, and disease duration as covariates. For glymphatic system analyses, linear mixed models were applied to DTI-ALPS scores in both hemispheres, considering group, time, their interaction, and covariates. A mediation analysis explored whether altered glymphatic scores mediated the relationship between PD phenotype and motor, cognitive, and psychiatric impairments due to previous evidence suggesting the role of glymphatic alterations in the accumulation of toxic substances [26, 27, 43, 53]. 

#### Genetic variables

SNP associations with body-first and brain-first PD phenotypes were tested, adjusting for age, and sex. Permutation testing with 5000 iterations controlled the false discovery rate with a threshold of 0.05 for statistical significance. Linear mixed models examined SNP effects on progression in motor, cognitive, and psychiatric domains in prodromal cases, including SNP variant group, time, interaction between group and time, age, sex, and education as fixed effects, along with random intercepts.

#### Unsupervised learning

The two latent space components derived from the VAE model representing the strongest patterns in the data were compared across different phenotypes. Statistical differences between phenotypes on these latent representations were assessed using Student’s t-test.

## Results

### Baseline motor and non-motor deficits are more severe in both clinical and prodromal body-first PD

**Fig. 1:**
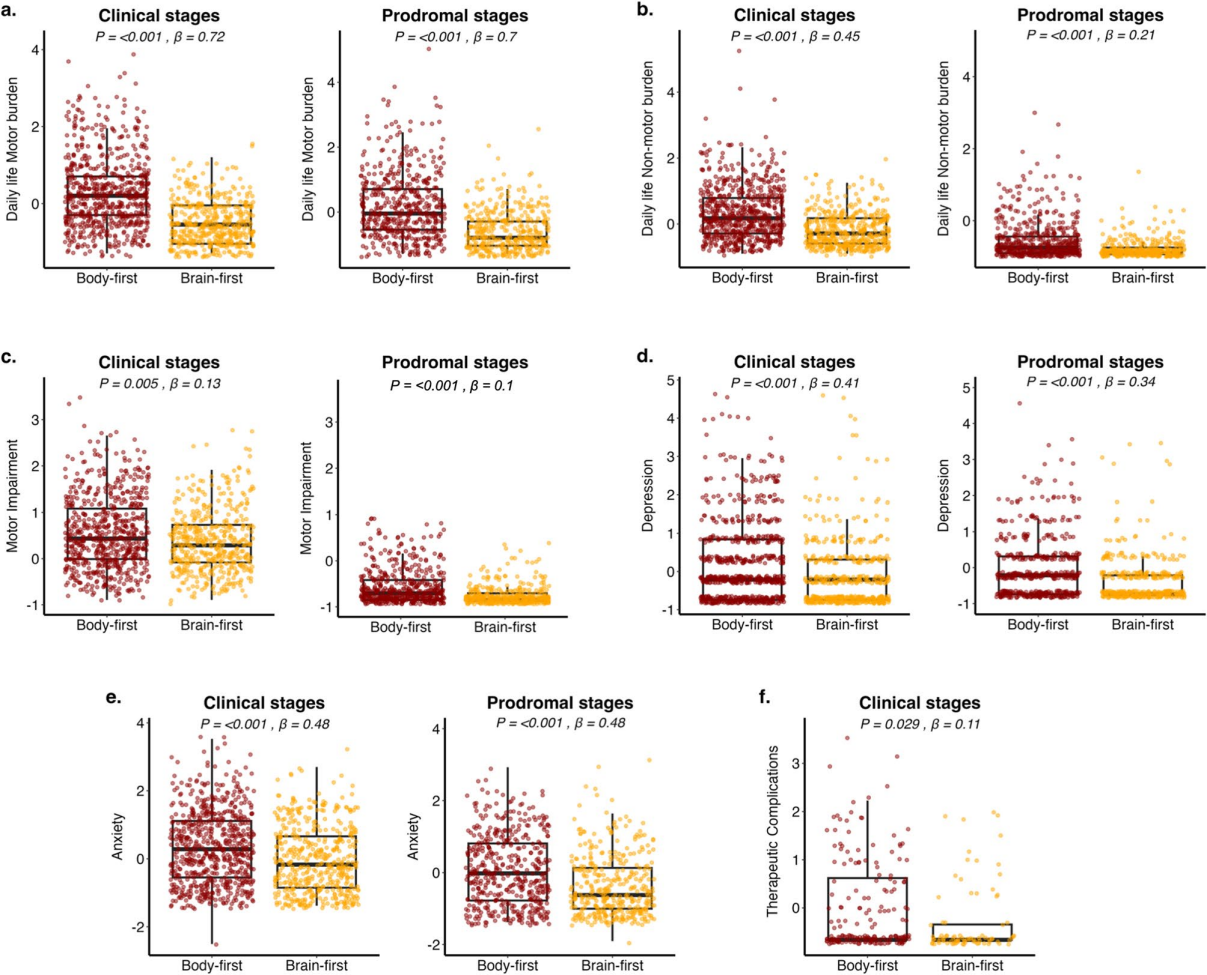
Baseline clinical differences between body-first and brain-first clinical and prodromal PD. Results from linear mixed effect models showing differences between body-first and brain-first clini-cal groups, and between body-first and brain-first prodromal groups. Plots are shown in pairs of clinical and prodromal groups for the same clinical outcome to highlight the similarities across clinical and prodromal stages of the same phenotype. The only plot that is not paired and is only shown for clinical cases is the one for complications of therapy, which was not assessable for prodromal cases as they were not receiving treatment. FDR is used to assess statistical significance. (**a**) Daily life motor burden, (**b**) Daily life non-motor burden, (**c**) motor impairment, (**d**) depression, (**e**) anxiety, (**f**) therapeutic complications.

Compared to brain-first PD, body-first patients were older both at enrolment (*p* < 0.001) and at diagnosis (*p* < 0.001), had a longer disease duration (*p* < 0.001), and had a greater diagnostic delay (*p* = 0.008) (Table 1). At baseline, we found that PD patients with a body-first phenotype experienced greater impairment in daily living activities (UPDRS I, β = 0.72, *p* < 0.001; UPDRS II, β = 0.45, *p* < 0.001), motor function (UPDRS III, β = 0.13, *p* = 0.005), and had more frequent therapy-related complications (UPDRS IV, β = 0.11, *p* = 0.029) compared to those with brain-first PD, after adjusting for age, sex, education, and disease duration (Fig. 1, Table S2). Specifically, body-first PD patients exhibited worse bradykinesia (β = 0.18, *p* = 0.001), axial symptoms (β = 0.16, *p* = 0.001), lower limbs’ disturbances (β = 0.14, *p* = 0.011), and posture and gait impairment (β = 0.13, *p* = 0.012) (Table S2). Additionally, these patients also had higher levels of depression (GDS, β = 0.41, *p* < 0.001) and anxiety (STAI, β = 0.48, *p* < 0.001).

**Table 1:** Demographic and clinical characteristics of body-first and brain-first PD at clinical and prodromal stages at baseline

	Clinical Stage			Prodromal Stage		
	Body-first (*n* = 667)	Brain-First (*n* = 460)	*p*-value	Body-first (*n* = 519)	Brain-First (*n* = 391)	*p*-value
Age, years	65.00 [57.90, 70.27]	61.55 [54.50, 68.20]	< 0.001	66.20 [61.68, 71.10]	63.10 [58.50, 68.10]	< 0.001
Sex (M), n (%)	404 (60.6)	285 (62.0)	0.664	298 (57.4)	154 (39.4)	< 0.001
Education, years	16.00 [14.00, 18.00]	16.00 [14.00, 18.00]	0.789	17.00 [16.00, 19.00]	17.00 [16.00, 18.00]	0.930
Disease duration, years	0.59 [0.17, 1.83]	0.41 [0.17, 1.17]	< 0.001	NA	NA	NA
Age at onset, years	63.50 [55.94, 69.06]	60.79 [53.17, 67.08]	< 0.001	NA	NA	NA
Diagnosis delay	1.00 [0.50, 2.00]	0.83 [0.42, 1.75]	0.022	NA	NA	NA
**Clinical Scales**						
UPDRS						
I	6.00 [4.00, 8.00]	3.00 [1.00, 4.00]	< 0.001	5.00 [3.00, 8.00]	2.00 [1.00, 4.00]	< 0.001
II	7.00 [4.00, 11.00]	4.00 [2.00, 6.00]	< 0.001	1.00 [0.00, 3.00]	0.00 [0.00, 1.00]	< 0.001
III	21.00 [15.00, 30.00]	19.00 [13.75, 25.00]	< 0.001	3.00 [1.00, 6.00]	1.00 [0.00, 4.00]	< 0.001
IV	0.00 [0.00, 4.00]	0.00 [0.00, 1.00]	0.008	NA	NA	NA
MoCA	27.00 [25.00, 29.00]	27.00 [25.00, 29.00]	0.540	27.00 [25.00, 28.00]	27.00 [25.00, 29.00]	0.007
ESS	6.00 [4.00, 9.00]	4.00 [2.00, 7.00]	< 0.001	6.00 [4.00, 8.50]	4.00 [3.00, 6.00]	< 0.001
RBDSQ	6.00 [4.00, 8.00]	2.00 [1.00, 3.00]	< 0.001	7.00 [5.00, 11.00]	2.00 [1.00, 3.00]	< 0.001
SCOPA-AUT	13.00 [10.00, 18.00]	6.00 [4.00, 8.00]	< 0.001	13.00 [8.00, 16.00]	6.00 [3.00, 7.00]	< 0.001
GDS	6.00 [5.00, 8.00]	5.00 [5.00, 7.00]	< 0.001	6.00 [5.00, 7.00]	5.00 [5.00, 6.00]	< 0.001
STAI	62.00 [51.00, 73.00]	55.00 [46.00, 66.00]	< 0.001	58.00 [48.00, 69.00]	50.00 [45.00, 60.00]	< 0.001
**Genetics**						
GBA, n (%)	65 (9.7)	24 (5.2)	0.387	78 (15.0)	111 (28.4)	< 0.001
LRRK2, n (%)	118 (17.7)	55 (12.0)	0.928	94 (18.1)	133 (34.0)	< 0.001
PRKN, n (%)	5 (1.0)	6 (1.7)	0.394	0 (0.0)	0 (0.0)	NA
PINK1, n (%)	1 (0.2)	0 (0.0)	NA	0 (0.0)	0 (0.0)	NA
SNCA, n (%)	18 (2.7)	11 (2.4)	0.535	3 (0.6)	7 (1.8)	0.109

Data are reported as median [interquartile range] and n (%). *p*-values are obtained from Wilcoxon rank-sum test (Mann-Whitney U) for continuos variables, and Chi-square test for categorical. For genetic comparisons, we performed logistic regression with PD phenotypes as outcome, and genetic groups as predictors. Age sex education and disease duration in clinical stages as covariates. Abbreviations: UPDRS: Unified Parkinson’s disease rating scale; MoCA: Montreal Cognitive Assessment; ESS: Epworth Sleepiness Scale; RBDSQ: REM sleep behavior disorder Questionnaire; SCOPA-AUT: Scale for Outcomes in Parkinson’s disease– Autonomic; GDS: Geriatric Depression Scale; STAI: State-Trait Anxiety Inventory. *GBA* (Glucosylceramidase beta), *LRRK2* (Leucine-Rich Repeat Kinase 2), *PRKN* (Parkin RBR E3 Ubiquitin Protein Ligase), *PINK1* (PTEN-Induced Kinase 1), and *SNCA* (alpha-synuclein)

Interestingly, similar results were observed in the early prodromal cohort, where body-first cases performed worse than brain-first cases on the same tests as their clinically diagnosed counterparts (daily living activities: UPDRS I, β = 0.7, *p* < 0.001; UPDRS II, β = 0.21, *p* < 0.001; motor function: UPDRS III, β = 0.1, *p* < 0.001; bradykinesia, β = 0.09, *p* < 0.001; axial symptoms, β = 0.06, *p* = 0.017; lower limbs’ disturbances, β = 0.06, *p* = 0.012; and posture and gait impairment, β = 0.06, *p* = 0.050; depression: GDS, β = 0.34, *p* < 0.001; and anxiety: STAI, β = 0.48, *p* < 0.001). We have to consider that therapy-related complications (UPDRS IV) could not be compared between prodromal cases, as they do not receive any PD medication (Fig. 1, Table S3). Additionally, prodromal body-first had worse tremor (β = 0.1, *p* = 0.001) and upper limbs’ signs (β = 0.13, *p* < 0.001) at baseline. To determine if these results were driven solely by the presence of REM sleep disturbances, which have been previously associated with worse prognosis in ageing and PD [54] we reclassified PD patients into brain-first and body-first phenotypes based only on autonomic symptoms. These analyses yielded similar results, indicating that body-first PD is not synonymous with REM sleep behaviour disorder (Table S4-5). Supplementary analyses including additional confounders, such as H&Y PD stage, cardiovascular comorbidities, and treatment with a drug that could mask RBD, confirmed our findings at both prodromal and clinical stages. These analyses showed that the worse disease progression in the body-first group was not driven by more severe disease at enrolment or higher comorbidity burden (Table S6-S7).

Altogether, these findings support the hypothesis that body-first PD has distinct clinical features that are detectable since the prodromal stages of the disease.

### Longitudinal clinical progression is accelerated in both clinical and prodromal body-first PD

**Fig. 2:**
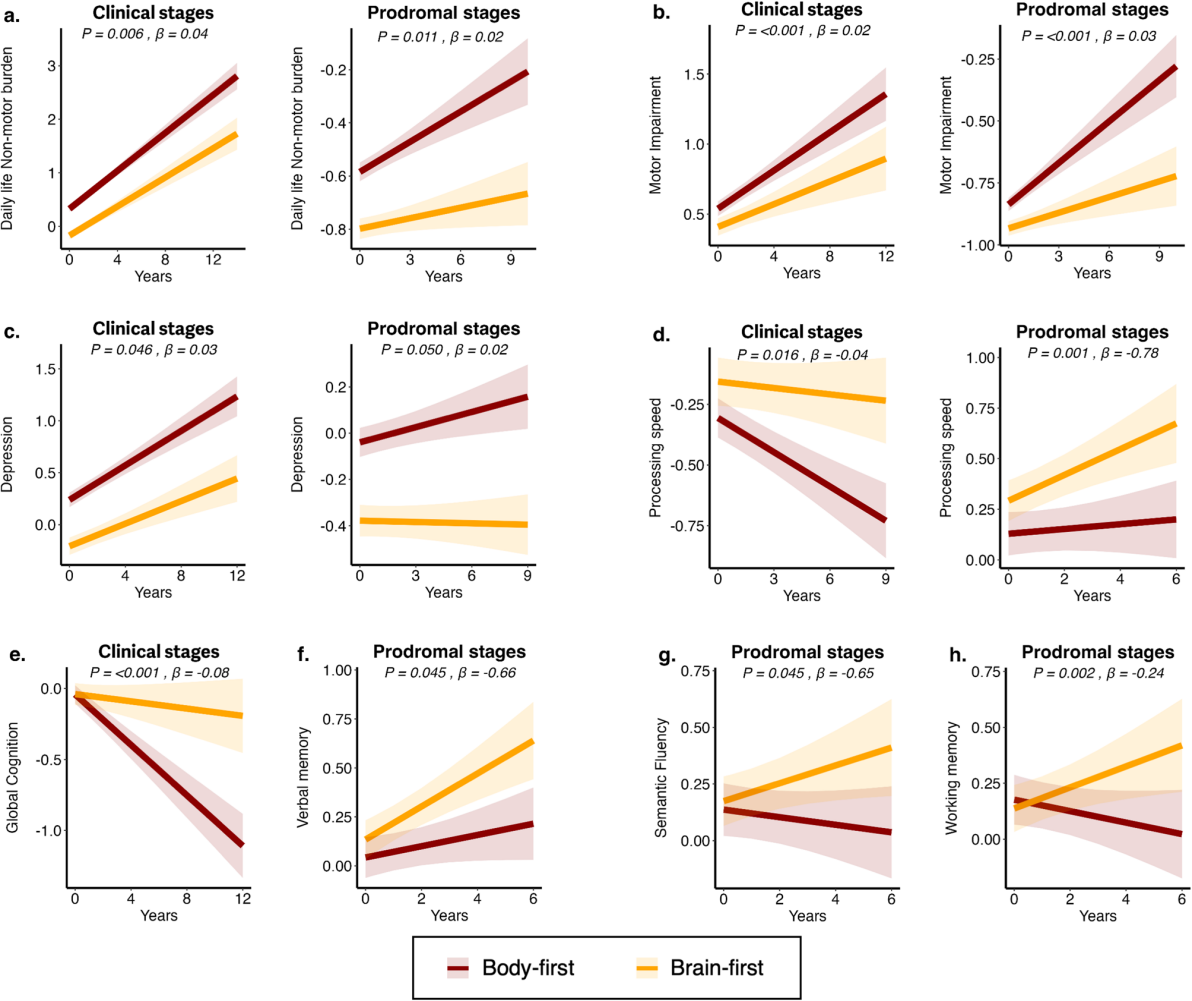
Longitudinal clinical trajectories in body-first and brain-first clinical and prodromal PD. Results from linear mixed effect models showing differences in clinical trajectories between body-first and brain-first clinical groups, and between body-first and brain-first prodromal groups. Plots are shown in pairs of clinical and prodromal groups for the same clinical outcome to highlight the similarities across clinical and prodromal stages of the same phenotype. FDR is used to assess statistical significance. (**a**) Daily life non-motor burden, (**b**) motor impairment, (**c**) depression, (**d**) processing speed, (**e**) global cognition, (**f**) verbal memory, (**g**) semantic fluency, (**h**) working memory.

Consistent with our baseline findings, longitudinal analyses revealed that body-first PD had a faster decline in both non-motor (UPDRS II, β*time = 0.04, *p* = 0.006 ) and motor (UPDRS III, β*time = 0.02, *p* < 0.001) functions, with worse progression of axial (β*time = 0.04, *p* = 0.031) and postural-gait impairment (β*time = 0.06, *p* = 0.007), worse complications due to therapy (UPDRS IV, β*time = 0.03, *p* < 0.001) as well as worse depression (GDS, β*time = 0.03, *p* = 0.046), compared to brain-first PD (Fig. 2, Table S2). Additionally, these patients showed a more pronounced decline in global cognition (MoCA, β*time = -0.08, *p* < 0.001) and attention (SDMT, β*time = -0.04, *p* = 0.016), with a higher risk of developing PD dementia in body-first PD (HR, 95% CI: 2.15, 1.45–3.19; *p* < 0.001; Figure S1) using a MoCA cut-off of 21, suggesting a clearer impairment of cognitive abilities as the disease progressed within this group. We confirmed these findings also when we identified body-first using only autonomic symptoms (Table S4) and after correcting for additional confounders (Table S6). Similarly, prodromal body-first cases showed a longitudinal decline in non-motor (UPDRS II, β*time = 0.02, *p* = 0.011) and motor (UPDRS III, β*time = 0.03, *p* < 0.001) functions, including bradykinesia (β*time = 0.03, *p* = 0.003), rigidity (β*time = 0.02, *p* = 0.003), and axial (β*time = 0.03, *p* = 0.003) and postural-gait impairment (β*time = 0.03, *p* = 0.013). These cases also exhibited worse cognitive abilities in attention (SDMT, β*time = -0.78, *p* = 0.001), executive functions (SF, β*time = -0.65, *p* = 0.045; LNS, β*time = -0.24, *p* = 0.002), and memory (Recall HVLT, β*time = -0.66, *p* = 0.045) over time (Fig. 2, Table S2). These findings indicate that the longitudinal clinical changes observed in clinical stages in body-first are largely present in prodromal stages and were also confirmed when body-first prodromal cases were identified using only autonomic symptoms (Table S5), and after correcting for additional confounders (Table S7).

**Fig. 3:**
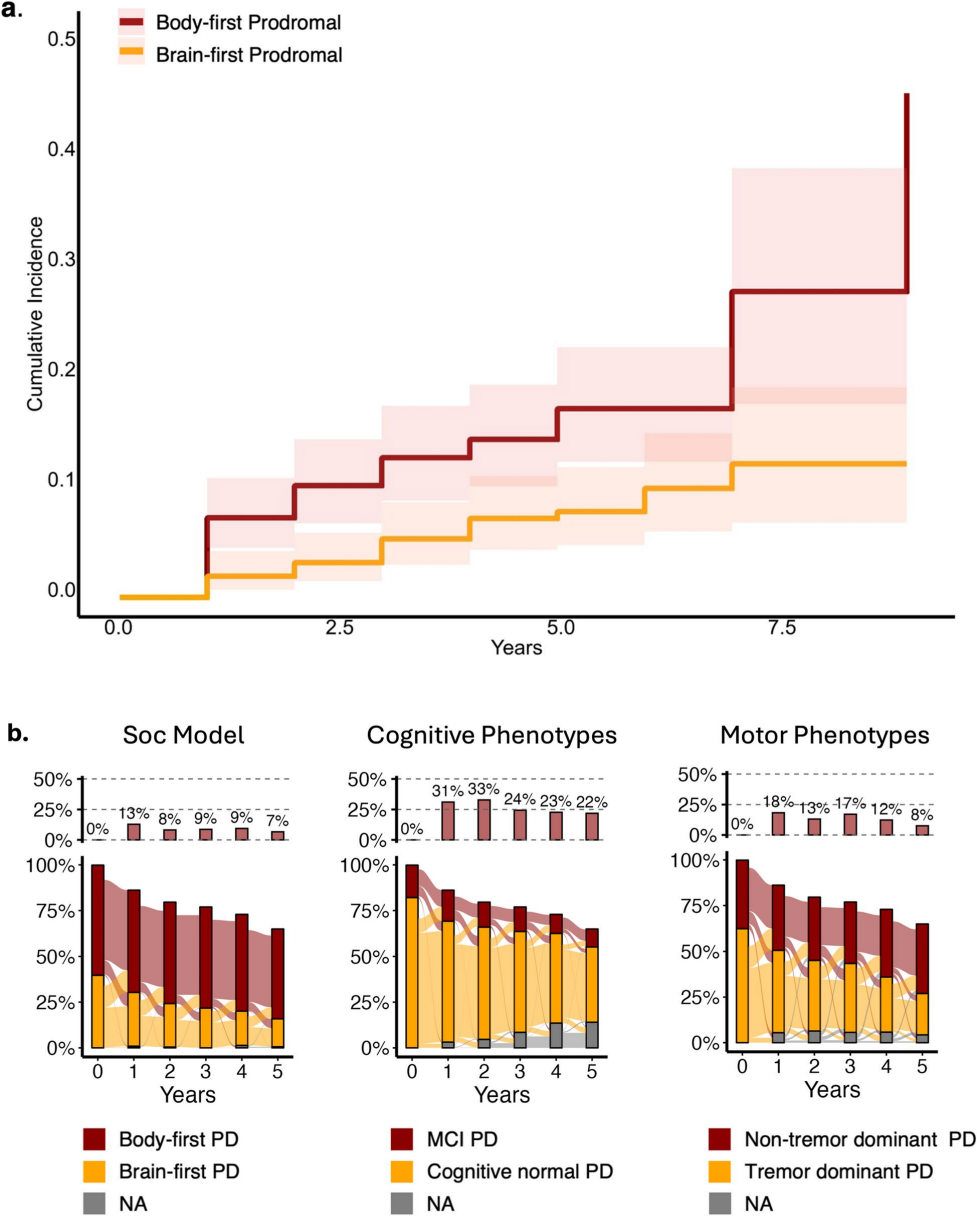
Conversion to PD in prodromal cases and stability of different PD phenotypes. Results from Kaplan-Maier curves demonstrate a higher cumulative incidence of Parkinson´s Disease (PD) in body-first compared to brain-first prodromal cases, shaded areas represent confidence intervals of cumulative incidence (**a**). Percentage (%) of cases classified as body-first or brain-first (SOC Model), mild cognitive impairment (MCI) or cognitively normal (cognitive phenotypes), non-tremor dominant or tremor dominant (motor phenotypes) over a period of 5 years (**b**). In the upper panel are reported the percentages of incorrect reclassification (shifts from worse prognosis to better prognosis phenotype) at each time point. The percentage proportion of body-first shifting to brain-first is lower compared to MCI shifting to cognitively normal, and non-tremor dominant shifting to tremor-dominant. PD: Parkinson’s Disease; SOC Model: The Synuclein Origin and Connectome Model

Beyond its association with worse disease progression across multiple domains, a body-first phenotype predicted a higher risk of conversion to PD in prodromal cases (HR: 2.02, 1.15–3.55; *p* = 0.014, Fig. 3a). These results remained significant after correcting for comorbidities and risk factors as well as excluding the potential confounding effect of medication that could mask RBD symptoms (HR: 1.78, 1.03–3.08; *p* = 0.039). Notably, these analyses could not be conducted using other PD classifications, such as MCI/cognitively normal or non-tremor dominant/tremor dominant, that do not allow any classification in prodromal stages due to the lack of significant cognitive or motor deficits at baseline in prodromal cases.

Furthermore, considering emerging evidence showing instability of PD classifications with patients often transitioning between subtypes over time [55, 56] we detected high degrees of instability in traditional PD classification systems: 24.4% and 21.9% of cases changed from MCI to cognitively normal after 3 and 5 years, whereas 17.1% and 7.5% cases changed from non-tremor to tremor-dominant after 3 and 5 years. Relative to established classifications, our analyses indicated that the classification based on body-like or brain-like symptoms offers a more stable and reliable approach to predicting disease progression. Specifically, a lower proportion of cases shifted from body-first to brain-first over 3 years (8.6%) and 5 years (6.7%) compared to shifts observed in other classification systems: (Fig. 3b). These results highlight the robustness of the body-first versus brain-first approach for PD prognosis.

### Locus coeruleus connectivity patterns differ between body-first and brain-first cases

**Fig. 4:**
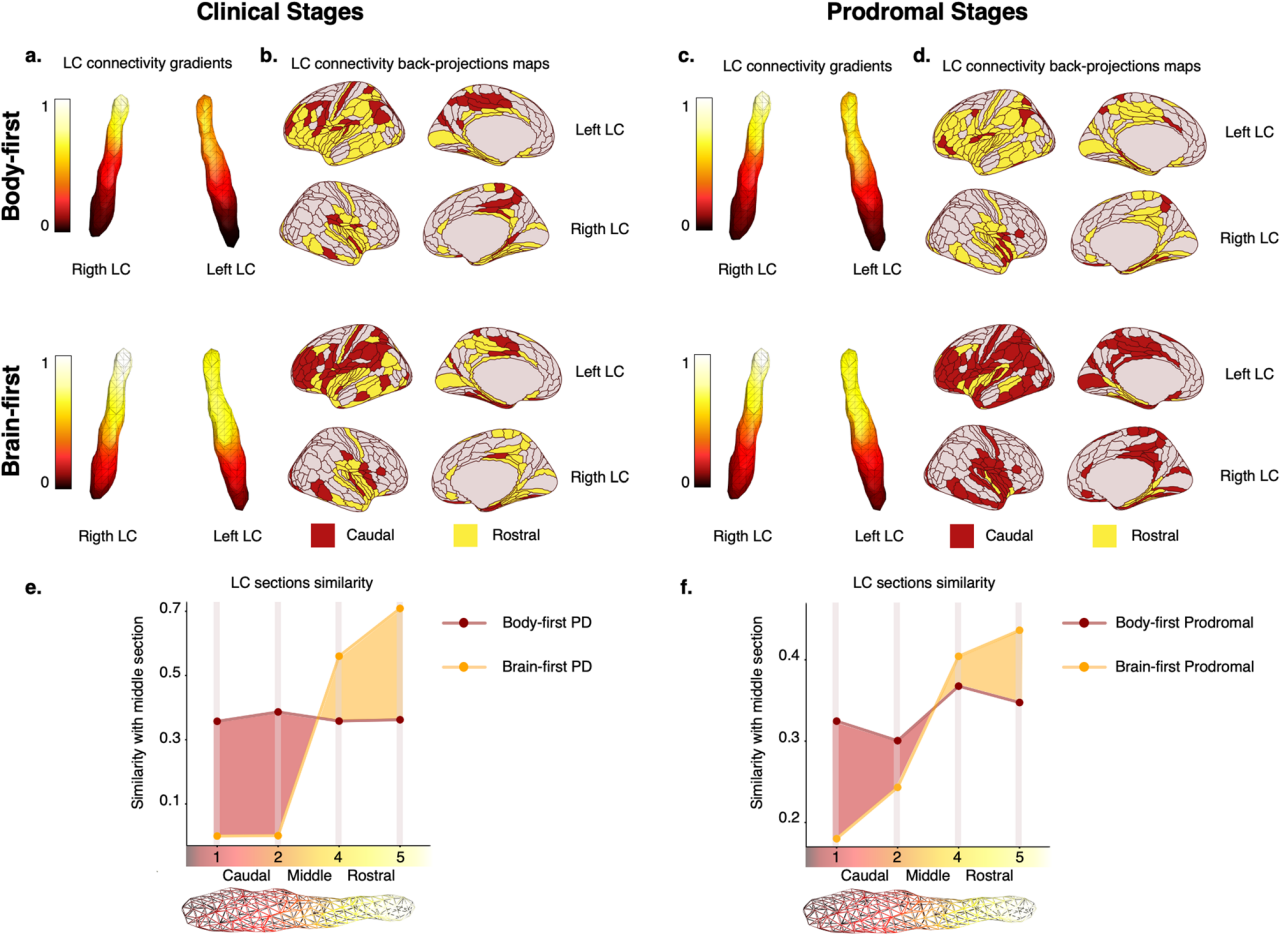
Locus Coeruleus (LC) connectivity gradients in body-first and brain-first clinical and prodromal PD. Significant differences in LC spatial features between body-first (upper row) and brain-first (middle row) Parkinson’s disease (PD). An extension of caudal-like connectivity of the LC (dark red, lower part of the gradient) was found in body-first clinical (**a**) and prodromal (**c**) stages, whereas the extension of rostral LC connectivity (yellow, higher part of the gradient) was found in brain-first clinical (**b**) and prodromal (**d**) stages. When projecting the LC connections on the cortical surface, body-first cases showed fewer brain areas connected to the caudal LC part (dark red), whereas brain-first had fewer connections to the rostral part (yellow), supporting the distinct progression patterns of LC changes in both clinical (**b**) and prodromal (**d**) stages. The similarity plots assess how similar the rostral and caudal parts of the LC were to the middle part in functional connections. It depicts that the caudal part was more similar (dark red shaded area) in body-first clinical (**e**) and prodromal (**f**) stages, whereas in brain-first the rostral part was more similar (orange shaded area) both for clinical (**e**) and prodromal (**f**) stages. For clarity, e and f do not include the similarity of the middle section ($\:\text{i}=3$) with itself

The connectivity patterns of the LC showed a rostro-caudal gradient across groups, consistent with previous reports [28, 57, 58]. However, significant differences in LC spatial features were observed between body-first and brain-first PD, which were also present in the body-first and brain-first prodromal groups (Table S8). When we assessed how similar the rostral (superior) and caudal (inferior) parts of the LC were to the middle part of the LC in terms of functional connections, we observed that the caudal part of the LC was more similar to the middle part in body-first PD, whereas the rostral part was more similar in brain-first PD (Fig. 4). These patterns were replicated in prodromal cases (Fig. 4). When projecting these connections onto the cortical surface, body-first cases showed fewer brain areas connected to the caudal LC part, whereas brain-first had fewer connections to the rostral part, further supporting the distinct progression patterns of LC changes in these phenotypes (Fig. 4).

### Dopaminergic uptake is more asymmetric in brain-first PD patients

**Fig. 5:**
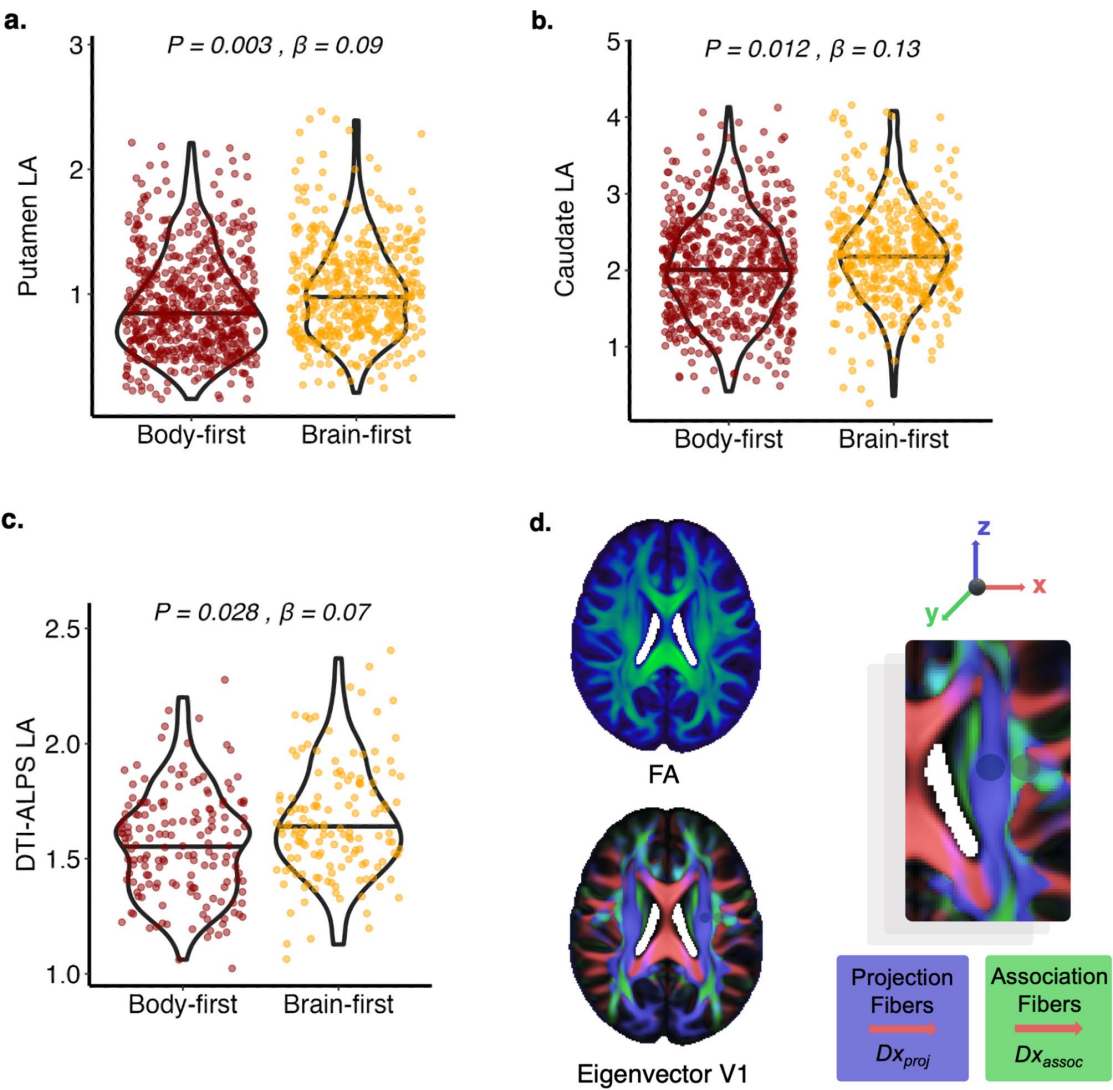
Differences in striatal dopaminergic binding and DTI-ALPS between body-first and brain-first PD. Results from linear mixed effect models showing significant differences between body-first and brain-first PD in the putamen (**a**) and caudate (**b**) of the hemisphere less affected (LA) by motor symptoms. Results from linear mixed effect models showing significant differences between body-first and brain-first PD in the glymphatic activity of the LA hemisphere (**c**). We show example images indicating the method applied to obtain the diffusivity along perivascular spaces (DTI-ALPS) according to the procedure detailed in the methods section. We generated color-coded fractional anisotropy and diffusivity maps (Dx, Dy, Dz) along the x-, y-, and z-axes. We placed spherical regions of interest on the lateral ventricle body (shaded areas), extracting mean diffusivity values from Dx, Dy, and Dz. The DTI-ALPS index was calculated using specific diffusivity measures: x-axis diffusivities in projection fibers (Dxproj) and association fibers (Dxassoc), as well as y-axis diffusivity in projection fibers (Dyproj) and z-axis diffusivity in association fibers (Dzassoc) [44] (**d**). Linear mixed-effect models were adjusted for age, sex, and disease duration, and corrected for FDR. PD: Parkinson´s Disease

Initial analyses of dopaminergic denervation in the putamen and caudate showed no significant differences between body-first and brain-first PD. However, when analysing the more and less affected hemispheres separately, we found that the more affected side had similar dopaminergic deficits in both phenotypes, while the less affected side had significantly higher binding in brain-first PD (β = 0.09, *p* = 0.003 for putamen; β = 0.13, *p* = 0.012 for caudate) (Fig. 5a-b), indicating greater dopaminergic asymmetry in this group. These observations were confirmed even after considering additional covariates as H&Y status, cardiovascular comorbidities and Clonazepam intake (β = 0.08, *p* = 0.008 for putamen; β = 0.11, *p* = 0.018 for caudate). Longitudinal analysis revealed no significant changes in dopaminergic binding in either hemisphere between the two PD subtypes, indicating that baseline differences diminished over time. Since dopaminergic imaging was unavailable for prodromal cases, we could not assess whether similar patterns were present in these early disease stages.

### Alterations in the glymphatic system are more asymmetric in brain-first PD

Similarly to the dopaminergic analyses, we only found differences in the glymphatic system (assessed through the DTI-ALPS index) in the less affected hemisphere, which showed significantly higher values indicating lower impairment in brain-first PD (β = 0.03, *p* = 0.028, Fig. 5c). This result is in line with a higher brain asymmetry present in this group. Mediation analyses showed that glymphatic alterations were a partial mediator of the effect between PD phenotype and motor impairment (10.5% mediation, *p* = 0.036), particularly in the less affected side (14.9% mediation, *p* = 0.002), indicating that greater glymphatic dysfunction in body-first PD contributed to the worse motor symptoms.

### Genetic differences between body-first and brain-first cases predict clinical progression in prodromal stages

**Fig. 6:**
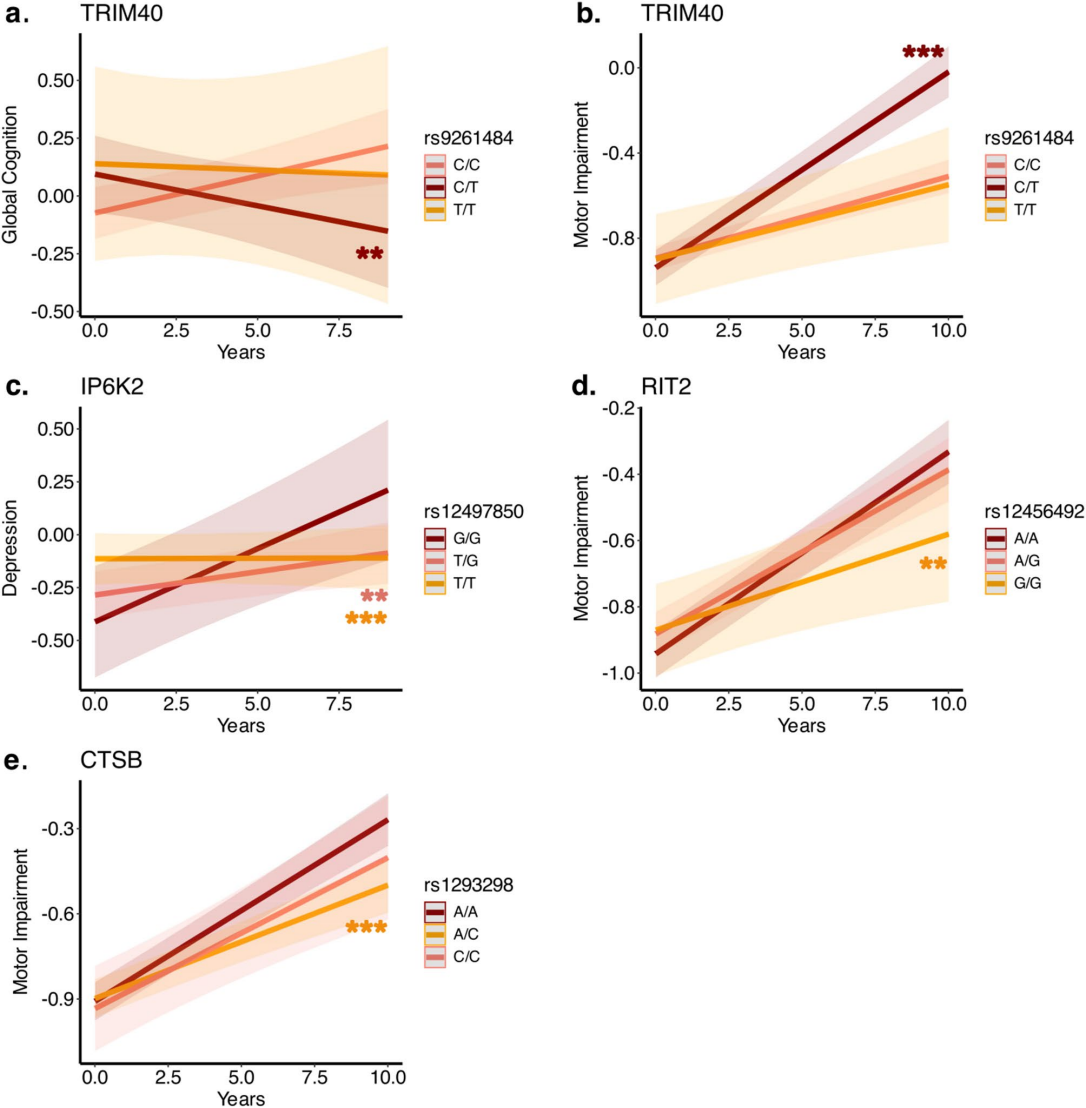
Differences between genetic polymorphisms and clinical progression in prodromal cases. Results from linear mixed-effect models showing the difference in clinical evolution between different genotype polymorphisms in prodromal Parkinson’s disease (PD) stages. The asterisks represent the adjusted *p*-value of the genotype x time interaction: **: <0.01; ***: <0.001. The heterozygous polymorphism (C/T) of tripartite motif-containing protein 40 (*TRIM40*) was associated with worse global cognition (**a**) and motor impairment (**b**); the homozygotic G allele of inositol heakisphosphate kinase 2 (*IP6K2*) was associated with the development of worse depression (**c**); the homozygotic G allele of Ras like without CAAX 2 (*RIT2*) was associated with reduced longitudinal motor impairment (**d**); and the heterozygous alleles (A/C) of cathepsin B (*CTSB*) were associated with lower motor impairment in prodromal PD stages (**e**)

To identify genetic markers distinguishing body-first from brain-first PD phenotypes, we investigated 123 SNPs previously associated with PD in GWAS studies. After adjusting for multiple comparisons, the Tripartite Motif Containing 40 (*TRIM40*, rs9261484 -T allele) and Golgi Brefeldin A resistant guanine nucleotide exchange Factor 1 (*GBF1*, rs10748818-G allele) were associated with a higher risk of developing brain-first PD (*p* < 0.05 for both, Table S9), while the Inositol Hexakisphosphate Kinase 2 (*IP6K2*, rs12497850-G allele), Ras-Like Without CAAX (*RIT2*, rs12456492-G allele), and Cathepsin B (*CTSB*, rs1293298-C allele) were associated with a lower risk of brain-first PD (*p* < 0.05 for all, Table S9). Further analysis in prodromal cases revealed that subjects with *TRIM40* heterozygous alleles (C/T) alleles experienced faster motor and cognitive decline, while those with *IP6K2* homozygous G alleles showed greater depression over 12 years in prodromal cases. Conversely, subjects with RIT2 homozygous G allele and CTSB heterozygous alleles (A/C) demonstrated slower motor impairment (Fig. 6, Table S10). These findings suggest that genes linked to body-first and brain-first PD phenotypes influence clinical progression in prodromal cases.

### Unsupervised deep-learning analysis identifies body-first and brain-first phenotypes

**Fig. 7:**
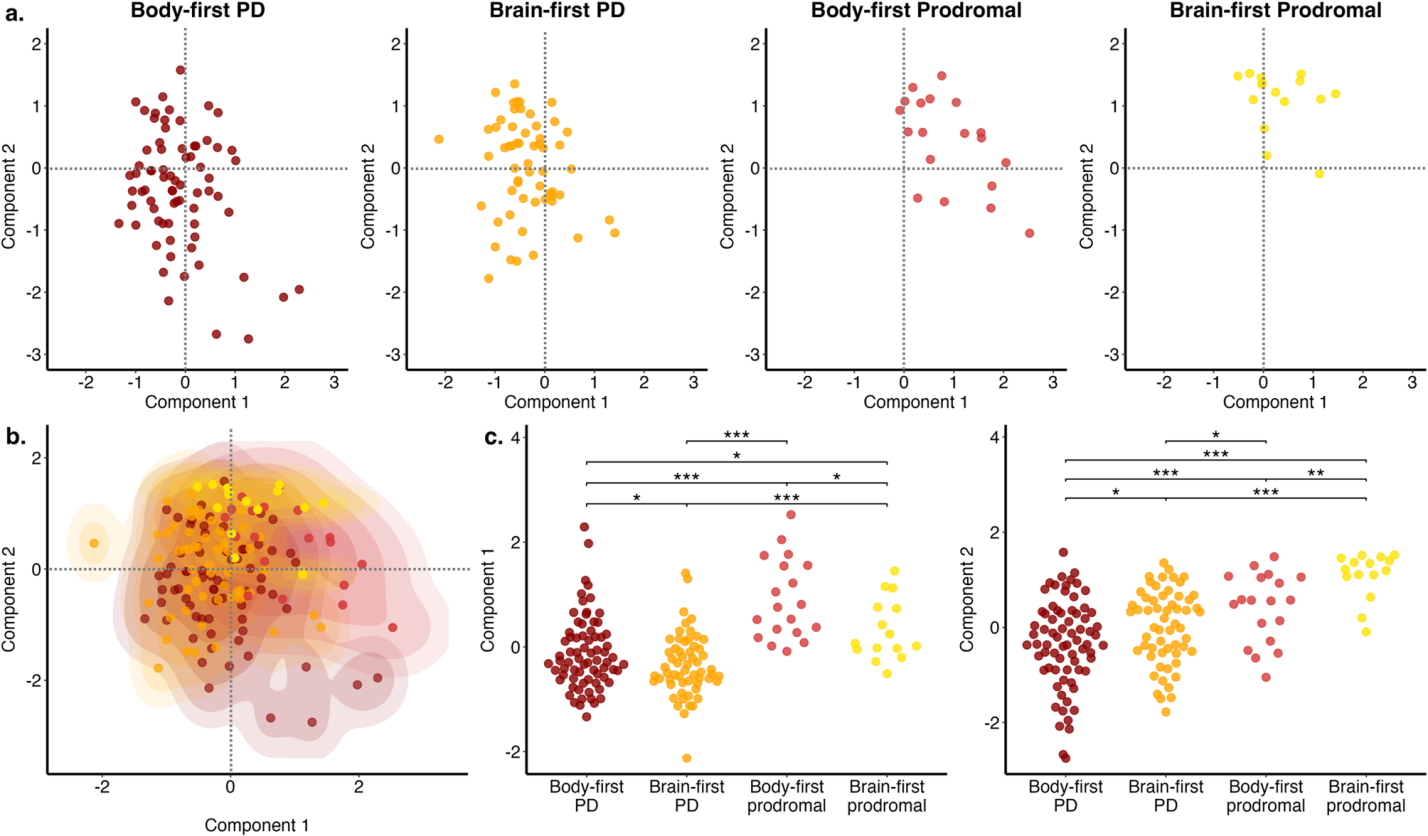
Unsupervised deep-learning analysis of data-driven phenotypes in clinical and prodromal stages of PD. Results from variational autoencoders (VAE) showing the different mapping of Parkinson’s disease (PD) phenotypes across the latent space for input data encompassing clinical, imaging, and genetic features. Distribution of subjects in the latent space demonstrating the different spatial distribution due to phenotype and clinical state, i.e., clinical diagnosed PD and prodromal cases (**a**). Brain-first phenotypes tend to be placed in the upper portion of the latent space, while body-first phenotypes tend to be placed in the bottom portion (**b**). Statistical comparisons between groups show significant differences between all phenotypes along both latent space components (**c**). The latent space component 1 distinguished the phenotypes between clinical and prodromal stages, while the latent space component 2 distinguished between body-first and brain-first phenotypes across different disease stages

Using a VAE, we mapped patient data into a 2D latent space, where each point represents a patient’s phenotype described by baseline clinical, imaging, and genetic features. Patients with similar latent space coordinates shared similar characteristics (Fig. 7a). Figure 7b shows how different phenotypes are distributed using kernel smoothing for probability estimation. Brain-first phenotypes tend to be placed in the top part of the latent space, while body-first phenotypes tend to appear in the bottom part. Moving from left to right across the plot, the severity of the clinical condition increases, progressing from early, prodromal stages to clinical PD. The latent space revealed distinct regions for body-first and brain-first phenotypes in both clinical and prodromal cases, with significant differences across both dimensions (Fig. 7c). This unsupervised clustering strongly aligns with and validates the body-first and brain-first framework, effectively distinguishing phenotypic variations in both clinical and prodromal stages.

## Discussion

The increasing recognition that PD is a heterogeneous disorder has led to the identification of different PD subtypes using various clinical and imaging measures. However, results across studies have been largely inconsistent, often reporting a varying number of clinical subtypes with different characteristics with limited applicability in clinical practice or trial design [4, 13, 59, 60]. This is particularly notable in data-driven studies, which vary in statistical methods and included variables, making it difficult to reach conclusions that can be generalized to other cohorts, due to the lack of a clear hypothesis [61, 62]. In this study, we provide longitudinal evidence supporting the existence of two clinicopathological phenotypes, body-first and brain-first, which were reproducible across clinical and prodromal stages of PD. These phenotypes were more stable and robust than other clinical classifications, predicting conversion to PD in prodromal stages, and conversion to PD dementia in clinical stages using a previously established cut-off [52]. Additionally, they showed brain imaging changes consistent with a progression of pathology from caudal to rostral regions in body-first and from rostral to caudal areas in brain-first cases, and they were associated with genetic markers linked to gut barrier disruption, inflammation, and α-synuclein aggregation. Finally, we were able to replicate these phenotypes using a self-supervised, deep-learning analysis, indicating that our approach combines the unbiased robustness of data-driven approaches with the theoretical consistency of hypothesis-driven methods. Altogether, these findings indicate that a pathologically-based definition of PD phenotypes is not only feasible and robust but also clinically valuable, suggesting it could be an important key to understanding PD’s heterogeneity, progression, and underlying biological mechanisms.

In the past few years, there has been a growing effort to establish a biological classification for PD that allows early detection and identification of different disease stages [1, 5]. To be useful, such a classification would require that markers that reflect underlying pathological changes (1) can be used not only in clinical but also in prodromal stages of the disease, and (2) are able to inform about different patterns of disease progression that underlie the distinct clinical symptoms, identifying biological mechanisms as a target for disease-modifying therapies [60]. In this study, we provide evidence that such an approach might already be available.

### Clinical utility and stability of body-first and brain-first phenotypes across PD stages

To address the first point on usability in prodromal disease stages, we used widely available clinical tests to detect REM sleep disorder and autonomic deficits. These tests identified cases that fit with a body-first or brain-first phenotype in two longitudinal (one clinical and one prodromal) cohorts. The analysis of these phenotypes revealed remarkable similarities in clinical and prodromal cases with body-first symptoms: they had greater motor dysfunction, anxiety, and depression at baseline as well as worse longitudinal motor progression, attention decline, and depression compared to their brain-first counterparts. These findings were observed after adjusting for disease duration and H&Y stage in the clinical PD cohort, showing that the greater clinical severity we observed in body-first cases was not due to a more advanced disease stage. Importantly, these results were still observed when subjects were classified as being body-first using autonomic deficits alone, refuting the assumption that our observations were merely driven by REM sleep disturbances, which have been linked to worse prognosis and were used as the main feature to identify body-first PD in previous studies [25, 63–65]. Our results were also confirmed after accounting for comorbidities such as diabetes and other cardiovascular diseases, indicating they were not influenced by these factors. Hence, our study is the first to show these striking similarities across different stages of body-first compared to brain-first PD both at baseline and longitudinally, ensuring that they are not driven by sleep dysfunction. We also demonstrated that body-first PD had a higher risk of conversion to PD dementia. To further examine the clinical utility of these phenotypes, we demonstrated that they were stable over 5 years, in contrast to clinical phenotypes defined using motor (postural instability gait-predominant) and cognitive (mild cognitive impairment) symptoms. These later phenotyping strategies were additionally limited by the fact that they could not be applied to the prodromal cohort due to the lack of cases with significant motor and cognitive deficits in early disease stages. Thus, the body-first and brain-first phenotyping not only exhibits greater stability over time but is also applicable to the earliest stages of PD. Of note, prodromal cases with body-first symptoms had a higher risk of conversion to PD, highlighting the utility of these symptoms in identifying individuals with a poorer prognosis, who could benefit most from disease-modifying therapies, and on whom an earlier diagnosis would have the largest clinical impact. Additionally, unsupervised deep learning analysis validated the distinction between body-first and brain-first individuals across various clinical stages. This finding suggests that the two phenotypes remain distinct even when classification is performed in an unsupervised manner, reinforcing the robustness of our approach.

### Different in vivo pathology progression patterns in body-first and brain-first PD

Regarding the second point on different patterns of disease progression, we selected three imaging measures that reflect the spreading of pathology across Braak’s stages, anatomical axes, and symmetric/asymmetric patterns. Firstly, we examined the functional connectivity of the LC, which is affected by α-synuclein pathology in Braak’s stages I-II. Our findings showed that, in body-first PD, connectivity was more severely reduced in the caudal part of the LC (although the entire structure presented changes), while in brain-first PD, reductions were observed only in the rostral part. These patterns were consistent in both clinical and prodromal cases, suggesting that these changes can be detected across different disease stages. Secondly, we assessed dopaminergic uptake in the striatum, which reflects changes in Braak’s stages III-IV. At baseline, brain-first PD showed greater dopaminergic binding in one of the brain hemispheres compared to body-first PD. However, this difference disappeared over time, indicating that brain-first cases, which start with more asymmetry, become more symmetric as the disease progresses. Hence, these dopaminergic patterns suggest that pathology in body-first PD spreads more symmetrically, while brain-first PD likely originates in one hemisphere and spreads to the other with disease progression. Lastly, we evaluated glymphatic alterations using the DTI-ALPS index, which reflects pathological changes in Braak’s stages V-VI. We found that glymphatic activity was reduced in the more affected hemisphere of both phenotypes but was again significantly higher or more preserved in the less affected hemisphere of brain-first compared to body-first PD, supporting our dopaminergic results and reinforcing the notion of an asymmetric hemisphere origin of pathology in brain-first cases. It is worth mentioning that, according to the SOC-model, body-first patients are expected to be more symmetric than brain-first cases, but this should not be seen as a dichotomy between symmetry or asymmetry in the two phenotypes [25]. Of note, glymphatic alterations mediated the relationship between having body-first symptoms and worse motor impairment, supporting its role as a potential neurodegenerative mechanism linked to lower clearance of toxic substances in body-first PD.

The more severe clinical progression observed in the body-first subtype, affecting both motor and non-motor domains, may be explained by the early and widespread involvement of multiple systems, including the dopaminergic, noradrenergic, and glymphatic pathways, on a bilateral level. This diffuse pathology likely disrupts compensatory mechanisms, leading to a more aggressive disease course. In contrast, in brain-first PD, the initially limited spreading of pathology allows for a better function of other systems outside the dopaminergic one, resulting in more localized signs mainly involving motor domains and slower progression with the preservation of alternative motor networks.

### Phenotype-derived genetic markers that predict clinical progression are linked to specific biological signatures

To further explore the biological differences between the body-first and brain-first phenotypes, a genetic analysis was conducted focusing on specific SNPs. Previous studies have suggested that *LRRK2* variants are associated with the brain-first phenotype, while *GBA* variants are linked to the body-first phenotype, though these associations are inconsistently supported across the literature [64]. In our cohort, no significant differences were found in GBA and LRRK2 mutation carriers between the two clinical PD phenotypes (Table 1). Both mutation groups were more frequently associated with a brain-first phenotype in the prodromal cohort (Table 1). It is important to note that this distribution likely reflects PPMI recruitment criteria for prodromal cohorts based on high-risk genetic mutations or prodromal features such as RBD. This may result in a selection bias favouring a higher percentage of individuals without genetic mutation in the body-first prodromal group. When comparing the two mutations, there were no differences in the distribution across the two phenotypes. In this study, 123 PD-associated SNPs from GWAS were analyzed [46, 66]revealing five SNPs with significantly different distributions between the two phenotypes, four of which predicted future clinical decline in prodromal cases. Some of these results were led by heterozygous carriers, suggesting that complex regulatory mechanisms may play a role, especially for *TRIM40* [67–69]. The *TRIM40* gene, implicated in inflammatory bowel syndrome and gut barrier integrity disruption due to cortical F-actin retraction and fragmentation was associated with worse motor and cognitive outcomes. This suggests that gut barrier subversion may increase vulnerability to factors like toxins and pathogens, potentially leading to early α-synuclein aggregation [7, 9, 70]. The *IP6K2* gene was linked to worse clinical progression, particularly higher depression levels in prodromal cases, and is known for its neuroprotective role in preventing PTEN-induced kinase 1 (*PINK1*)-mediated mitophagy in the brain [71]. Additionally, the *CTSB* and *RIT2* genes were associated with worse motor symptoms, with studies showing that their inhibition promotes α-synuclein aggregation. Specifically, *CTSB* cleaves α-synuclein within its amyloid region, preventing fibril formation in lysosomes [72]. *CTSB* variants have also been identified as modifiers of risk and age at onset in *GBA*-associated PD [73]with catB (*CTSB* product) inhibition impairing autophagy and leading to the accumulation of lysosomal content [74]. The *RIT2* product is a small GTPase whose loss leads to substantia nigra pars compacta (SNc) cell death and sex-specific motor dysfunction in mice [75]. *RIT2* is also involved in modulating *LRRK2* kinase activity, crucial for lysosomal function and protection against α-synuclein neuropathology [76]. 

### Limitations and future perspectives

Some limitations should be recognized in our study. Firstly, our classification relied on clinical scores, which are widely available but can be affected by subjective bias. Specifically, the RBDSQ depends on self-reported symptoms, which may be influenced by patient recall, limited awareness, or overlap with other sleep disorders, potentially reducing specificity [18, 77, 78]. Similarly, SCOPA-AUT captures subjective autonomic symptoms that can vary greatly among individuals, may be influenced by medications or comorbidities, and might not fully reflect objective dysfunction [19, 79]. Despite these caveats, their ease of use, scalability, and ability to capture patient-reported outcomes make them valuable for large-scale studies and clinical practice. More objective assessments, such as polysomnography for RBD or Metaiodobenzylguanidine (MIBG) scintigraphy of the heart for autonomic dysfunction, as well as α-synuclein seeding assay to verify α-synuclein status, could have provided deeper insights into PD phenotypes features, but access to these tests in clinical settings is often challenging. Secondly, we lacked detailed information on the timing of RBD or autonomic symptoms relative to motor onset, that were employed in other studies [80–82]. This may have led to misclassification of some brain-first individuals with early RBD or autonomic involvement into the body-first group, potentially limiting the strength of our findings. However, it is worth noting that in longitudinal follow-up time points, the rate of reclassification errors was lower using our approach than others based on motor or cognitive symptoms, suggesting that most of the individuals were correctly classified at baseline. Our study employs a large cohort, also applying this classification at early disease stages. This enhances the generalizability and practical utility of our classification, particularly in real-world clinical practice where detailed premotor symptom timelines are often unavailable or may be subject to recall bias. The prodromal cases included in our study represent well-recognized conditions for PD but do not cover the full spectrum of prodromal PD conditions and present some differences in sex distribution with their clinical counterparts, limiting the generalizability of our findings. Additionally, our genetic analysis focused on previously identified SNPs, which may have overlooked other relevant SNPs specific to the body-first and brain-first phenotypes. Future GWAS studies should investigate these phenotypes in more detail to uncover additional genetic associations, together with a deeper exploration of genetic and molecular mechanisms, to clarify the complex genotype-phenotype relationships suggested by our findings.

## Conclusions

Despite these limitations, our findings highlight distinct patterns of disease progression in body-first and brain-first PD. Body-first PD is characterized by widespread changes in the caudal LC, more symmetrical degeneration in the striatum, and glymphatic dysfunction, consistent with Braak’s staging and the SOC model hypothesis. In contrast, brain-first PD shows more asymmetrical degeneration and alterations in the rostral LC, indicating top-down disease progression. These clinical and imaging patterns reflect the different biological origins of the phenotypes. Our study also identified genetic markers associated with gut barrier disruption and impaired cellular functions, providing new insights into the biological mechanisms underlying body-first and brain-first PD. These findings underscore the potential of these genetic markers to identify individuals at high risk of worse clinical outcomes and support the development of personalized therapies targeting the specific mechanisms of each phenotype. Overall, our study shows that body-first and brain-first phenotypes offer a reliable way to predict disease progression, demonstrating stability over time and potential for application in clinical practice. This approach could be valuable in clinical trials and provide a deeper understanding of PD pathology, paving the way for new disease-modifying treatments.

## Electronic supplementary material

Below is the link to the electronic supplementary material.

Supplementary Material 1

Supplementary Material 2

## Data Availability

Data used in the preparation of this article were obtained on [2023-05-31] from the Parkinson’s Progression Markers Initiative (PPMI) database (https://www.ppmi-info.org/access-data-specimens/download-data), RRID: SCR_006431. For up-to-date information on the study, visit http://www.ppmi-info.org. Access to PPMI data is granted upon registration and adherence to data use policies. Data are available upon reasonable request to the corresponding authors.

## Abbreviations

PD: Parkinson’s disease
SOC: Synuclein origin and connectome
LC: Locus coeruleus
RBD: REM sleep behavior disorder
GBA: Glucosylceramidase beta
LRRK2: Leucine-rich repeat kinase 2
SNCA: Synuclein alpha
PPMI: Parkinson progression marker initiative
UPDRS: Unified parkinson’s disease rating scale
MoCA: Montreal cognitive assessment
HVLT: R-hopkins verbal learning test-revised
LNS: Letter and number sequencing test
SDMT: Symbol digit modalities test
GDS: Geriatric depression scale
STAI: State-trait anxiety inventory
RBDSQ: RBD questionnaire
SCOPA: AUT-scale for outcomes in parkinson’s disease-autonomic
MCI: Mild cognitive impairment
fMRI: Functional magnetic resonance imaging
DaTSCAN: Dopamine transporter imaging
DWI: Diffusion-weighted imaging
DTI: ALPS-diffusion tensor imaging analysis along the perivascular space
SPECT: Single photon emission computed tomography
SBR: Specific binding ratio
FSL: FMRIB software library
MCFLIRT: Motion correction FMRIB’s linear image registration tool
BET: Brain extraction tool
ICA: AROMA-independent component analysis-based automatic removal of motion artifacts
MNI152: Montreal neurological institute 152
colFA: Color-coded fractional anisotropy
Dx, Dy, Dz: Diffusivity along x-, y-, and z-axes
GWAS: Genome-wide association study
SNPs: Single nucleotide polymorphisms
VAE: Variational autoencoder
ReLU: Rectified linear unit
ELBO: Evidence lower bound
TRIM40: Tripartite motif containing 40
GBF1: Golgi brefeldin a resistant guanine nucleotide exchange factor 1
IP6K2: Inositol hexakisphosphate kinase 2
CTSB: Cathepsin B
RIT2: Ras-like without CAAX 2
SNc: Substantia nigra pars compacta
PINK1: PTEN induced kinase 1
