# Correction: Clinical progression and genetic pathways in body-first and brain-first Parkinson’s disease

**DOI:** 10.1186/s13024-025-00885-2

**Published:** 2025-08-20

**Authors:** Massimiliano Passaretti, Daniel Veréb, Mite Mijalkov, Yu-Wei Chang, Hang Zhao, Blanca Zufiria-Gerbolés, Jiawei Sun, Giovanni Volpe, Natalia Rivera, Matteo Bologna, Joana B. Pereira

**Affiliations:** 1 Department of Clinical Neuroscience, Karolinska Institutet, K8 Klinisk neurovetenskap, K8 Neuro Pereira, 171 77, Stockholm, Sweden; 2 Department of Human Neurosciences, Sapienza University of Rome, Rome, Italy; 3 Department of Physics, University of Gothenburg, Gothenburg, Sweden; 4 Immunology and Respiratory Medicine Division, Department of Medicine Solna, Center for Molecular Medicine, Karolinska Institutet, Karolinska University Hospital, Karolinska University Hospital, Solna, Stockholm, Sweden; 5 Rheumatology Division, Department of Medicine Solna, Center for Molecular Medicine, Karolinska Institutet, Karolinska University Hospital, Karolinska University Hospital, Solna, Stockholm, Sweden; 6 IRCCS Neuromed, Pozzilli, Italy

**Correction: Molecular Neurodegeneration (2025) 20:74**

10.1186/s13024-025-00866-5

The original article has been updated to correct figure captions and order, reference citations, and inconsistent p-value formatting.

