# Supplementary material for: Clinical progression and genetic pathways in body-first and brain-first Parkinson’s disease
Source: Mol Neurodegener. 2025 Jun 20;20:74. doi: 10.1186/s13024-025-00866-5 (PMC12181842; doi:10.1186/s13024-025-00866-5)
Supplement: Supplementary file 2 — Supplementary Material 2 [file 13024_2025_866_MOESM2_ESM.docx]

**Supplementary material**

**Table S1 | Demographic and clinical characteristics of healthy controls at baseline**

|  | **Healthy Controls**  **(n= 263)** |
| --- | --- |
| Age, years | 63.20 [55.90, 69.70] |
| Sex (M), n (%) | 164 (62.4) |
| Education, years | 16.00 [14.00, 18.00] |
| Disease duration, years | NA |
| Age at onset, years | NA |
| Diagnosis delay | NA |
| **Clinical Scales** |  |
| UPDRS |  |
| I | 2.00 [1.00, 4.00] |
| II | 0.00 [0.00, 0.00] |
| III | 0.00 [0.00, 2.00] |
| IV | NA |
| MoCA | 28.00 [27.00, 29.00] |
| ESS | 5.00 [3.00, 7.00] |
| RBDSQ | 2.00 [1.00, 4.00] |
| SCOPA-AUT | 5.00 [3.00, 8.00] |
| GDS | 5.00 [5.00, 6.00] |
| STAI | 51.00 [44.00, 62.00] |

**Table S2 |** Difference in clinical outcomes between clinical stages PD phenotypes

| **Clinical stages body first vs. brain first phenotype** | **Baseline** | | | **Longitudinal** | | | |
| --- | --- | --- | --- | --- | --- | --- | --- |
|  | β | T value | Adj P | β*time | T value | Adj P |  |
| **Daily life burden** |  |  |  |  |  |  |  |
| UPDRS I | 0.72 | 15.57 | **<0.001** | 0.01 | 1.23 | 0.220 |  |
| UPDRS II | 0.45 | 10.78 | **<0.001** | 0.04 | 3 | **0.006** |  |
| **Motor impairment** |  |  |  |  |  |  |  |
| UPDRS III | 0.13 | 3.04 | **0.005** | 0.02 | 4.04 | **<0.001** |  |
| Bradykinesia | 0.18 | 3.97 | **0.001** | 0.03 | 2.23 | 0.106 |  |
| Rigidity | 0.11 | 2.04 | 0.084 | 0.01 | 0.83 | 0.809 |  |
| Tremor | -0.05 | -0.85 | 0.398 | -0.01 | -0.71 | 0.809 |  |
| Posture and gait impairment | 0.13 | 2.98 | **0.012** | 0.06 | 3.3 | **0.007** |  |
| Axial | 0.16 | 3.78 | **0.001** | 0.04 | 2.81 | **0.031** |  |
| Upper limbs | 0.11 | 2.28 | 0.068 | 0.01 | 1.11 | 0.801 |  |
| Lower limbs | 0.14 | 3.08 | **0.011** | 0.03 | 2.75 | **0.031** |  |
| UPDRS IV | 0.11 | 2.19 | **0.029** | 0.03 | 4.68 | **<0.001** |  |
| **Cognitive funtion** |  |  |  |  |  |  |  |
| MoCA | 0.04 | 0.7 | 1.000 | -0.08 | -5.54 | **<0.001** |  |
| Benton Judgment of Line Orientation | -0.06 | -0.92 | 1.000 | -0.01 | -0.79 | 0.863 |  |
| HVLT Total Recall | -0.05 | -0.81 | 1.000 | -0.02 | -1.3 | 0.621 |  |
| HVLT Recognition Discrimination | -0.1 | -1.45 | 0.930 | -0.01 | -0.76 | 0.863 |  |
| Semantic Fluency | 0.03 | 0.38 | 1.000 | -0.02 | -1.42 | 0.621 |  |
| Letter Number Sequencing | -0.04 | -0.57 | 1.000 | -0.02 | -1.66 | 0.485 |  |
| Symbol Digit Modalities | -0.1 | -1.5 | 0.930 | -0.04 | -3.02 | **0.016** |  |
| **Psychiatric symptoms** |  |  |  |  |  |  |  |
| Geriatric Depression Scale | 0.41 | 7.36 | **<0.001** | 0.03 | 2.28 | **0.046** |  |
| State-Trait Anxiety Index | 0.48 | 8.3 | **<0.001** | 0.01 | 0.59 | 0.554 |  |

β coefficients with T and p values are derived from linear mixed effect models; p-values are adjusted for FDR. Significant p-values are reported in bold. Abbreviations: UPDRS: Unified Parkinson's disease rating scale; MoCA: Montreal Cognitive Assessment; HVLT: Hopkins verbal learning test.

**Table S3** | Difference in clinical outcomes between prodromal stages PD phenotypes

| **Prodromal stages body first vs. brain first** | **Baseline** | | | **Longitudinal** | | |
| --- | --- | --- | --- | --- | --- | --- |
|  | β | *T value* | *Adj P* | β**time* | *T* | *Adj P* |
| **Daily life burden** |  |  |  |  |  |  |
| UPDRS I | 0.7 | 12.7 | **<0.001** | -0.01 | -0.51 | 0.611 |
| UPDRS II | 0.21 | 7.98 | **<0.001** | 0.02 | 2.79 | **0.011** |
| **Motor Impairment** |  |  |  |  |  |  |
| UPDRS III | 0.1 | 4.76 | **<0.001** | 0.03 | 4.02 | **<0.001** |
| Bradykinesia | 0.09 | 4.41 | **<0.001** | 0.03 | 3.46 | **0.003** |
| Rigidity | 0 | -0.1 | 0.922 | 0.02 | 3.51 | **0.003** |
| Tremor | 0.1 | 3.64 | **0.001** | 0 | 0.03 | 0.976 |
| Posture and gait impairment | 0.06 | 2.24 | **0.050** | 0.03 | 2.77 | **0.012** |
| Axial | 0.06 | 2.76 | **0.017** | 0.03 | 3.54 | **0.003** |
| Upper limbs | 0.13 | 5.04 | **<0.001** | 0.02 | 2.97 | **0.010** |
| Lower limbs | 0.06 | 2.98 | **0.012** | 0.03 | 3.05 | **0.010** |
| UPDRS IV | -0.04 | -1.27 | 0.210 | 0.02 | 1.73 | 0.084 |
| **Cognitive function** |  |  |  |  |  |  |
| MoCA | -0.25 | -1.62 | 0.525 | 0 | -0.05 | 1.000 |
| Benton Judgment of Line Orientation | -0.23 | -0.98 | 0.988 | -0.01 | -0.09 | 1.000 |
| HVLT Total Recall | -0.96 | -1.16 | 0.988 | -0.66 | -2.61 | **0.045** |
| HVLT Recognition Discrimination | -1.72 | -2.26 | 0.167 | -0.14 | -0.49 | 1.000 |
| Semantic Fluency (SFT) | -0.38 | -0.41 | 1.000 | -0.65 | -2.6 | **0.045** |
| Letter Number Sequencing (LNS) | 0.14 | 0.57 | 1.000 | -0.24 | -3.66 | **0.002** |
| Symbol Digit Modalities (SDM) | -1.55 | -1.83 | 0.406 | -0.78 | -3.94 | **0.001** |
| **Psychiatric symptoms** |  |  |  |  |  |  |
| Geriatric Depression Scale (GDS) | 0.34 | 7.09 | **<0.001** | 0.02 | 2.25 | **0.050** |
| State-Trait Anxiety Index (STAI) | 0.48 | 7.93 | **<0.001** | 0.02 | 1.74 | 0.082 |

β coefficients with T and p values are derived from linear mixed effect models; p-values are adjusted for FDR. Significant p-values are reported in bold. Abbreviations: UPDRS: Unified Parkinson's disease rating scale; MoCA: Montreal Cognitive Assessment; HVLT: Hopkins verbal learning test.

**Table S4** | Difference in clinical outcomes between clinical stages PD phenotypes**:** Autonomic dysfunction only vs. brain-first

| **PD clinical stages autonomic disfunction vs. brain first** | **Baseline** | | | **Longitudinal** | | |
| --- | --- | --- | --- | --- | --- | --- |
|  | β | *T value* | *Adj P* | β**time* | *T* | *Adj P* |
| **Daily life burden** |  |  |  |  |  |  |
| UPDRS I | 0.60 | 13.10 | **<0.001** | 0.04 | 2.71 | **0.014** |
| UPDRS II | 0.88 | 17.67 | **<0.001** | 0.00 | 0.41 | 0.681 |
| **Motor Impairment** |  |  |  |  |  |  |
| UPDRS III | 0.16 | 3.48 | **0.001** | 0.02 | 4.19 | **<0.001** |
| Bradykinesia | 0.21 | 4.38 | **<0.001** | 0.03 | 2.29 | 0.091 |
| Rigidity | 0.13 | 2.25 | **0.049** | 0 | 0.23 | 1.000 |
| Tremor | 0.07 | 1.07 | 0.286 | -0.01 | 0.58 | 1.000 |
| Posture and gait impairment | 0.17 | 3.41 | **0.003** | 0.09 | 4.18 | **<0.001** |
| Axial | 0.20 | 4.23 | **<0.001** | 0.06 | 3.44 | **0.004** |
| Upper limbs | 0.14 | 2.74 | **0.019** | 0.01 | 1.04 | 0.898 |
| Lower limbs | 0.16 | 3.23 | **0.005** | 0.04 | 3.00 | **0.015** |
| UPDRS IV | 0.18 | 3.27 | **0.001** | 0.03 | 3.59 | **<0.001** |
| **Cognitive function** |  |  |  |  |  |  |
| MoCA | -0.03 | -0.49 | 1.000 | -0.09 | -5.41 | **<0.001** |
| Benton Judgment of Line Orientation | 0.01 | 0.15 | 1.000 | -0.01 | -0.72 | 0.947 |
| HVLT Total Recall | 0.04 | 0.52 | 1.000 | -0.02 | -1.50 | 0.401 |
| HVLT Recognition Discrimination | 0.11 | 1.44 | 1.000 | -0.01 | -0.66 | 0.947 |
| Semantic Fluency (SFT) | -0.02 | -0.23 | 1.000 | -0.03 | -2.06 | 0.199 |
| Letter Number Sequencing (LNS) | 0.00 | -0.02 | 1.000 | -0.03 | -1.81 | 0.284 |
| Symbol Digit Modalities (SDM) | 0.09 | 1.25 | 1.000 | -0.05 | -3.69 | **0.002** |
| **Psychiatric symptoms** |  |  |  |  |  |  |
| Geriatric Depression Scale (GDS) | -0.53 | -8.63 | **<0.001** | 0.03 | -1.92 | 0.112 |
| State-Trait Anxiety Index (STAI) | -0.55 | -8.81 | **<0.001** | 0.01 | -0.66 | 0.512 |

β coefficients with T and p values are derived from linear mixed effect models; p-values are adjusted for FDR. Significant p-values are reported in bold. Abbreviations: UPDRS: Unified Parkinson's disease rating scale; MoCA: Montreal Cognitive Assessment; HVLT: Hopkins verbal learning test.

**Table S5** | Difference in clinical outcomes between prodromal stages PD phenotypes**:** Autonomic dysfunction only vs. brain-first

| **Prodromal stages autonomic disfunction vs. brain first** | **Baseline** | | | **Longitudinal** | | |
| --- | --- | --- | --- | --- | --- | --- |
|  | β | *T value* | *Adj P* | β**time* | *T* | *Adj P* |
| **Daily life burden** |  |  |  |  |  |  |
| UPDRS I | 0,68 | 10,19 | **<0.001** | 0,01 | 0,6 | 0,202 |
| UPDRS II | 0,19 | 7,18 | **<0.001** | 0,02 | 1,65 | 0,547 |
| **Motor Impairment** |  |  |  |  |  |  |
| UPDRS III | 0,09 | 3,69 | **<0.001** | 0,03 | 2,58 | **0,021** |
| Bradykinesia | 0,08 | 3,16 | **0,008** | 0,02 | 2,45 | 0,075 |
| Rigidity | 0,03 | 1,08 | 0,282 | 0,01 | 1,78 | 0,307 |
| Tremor | 0,09 | 2,37 | 0,074 | 0,00 | -0,49 | 0,908 |
| Posture and gait impairment | 0,06 | 1,75 | 0,242 | 0,02 | 1,68 | 0,307 |
| Axial | 0,05 | 1,75 | 0,242 | 0,02 | 2,82 | **0,036** |
| Upper limbs | 0,1 | 3,25 | **0,007** | 0,02 | 2,72 | **0,042** |
| Lower limbs | 0,09 | 3,31 | **0,007** | 0,01 | 0,75 | 0,908 |
| UPDRS IV | -0,03 | -1,38 | 0,169 | 0,03 | 2,16 | **0,032** |
| **Cognitive function** |  |  |  |  |  |  |
| MoCA | -0,14 | -2,09 | 0,225 | 0,00 | 0,06 | 1,000 |
| Benton Judgment of Line Orientation | 0,05 | 0,51 | 1,000 | -0,02 | -0,46 | 1,000 |
| HVLT Total Recall | -0,07 | -0,65 | 1,000 | -0,08 | -2,31 | 0,155 |
| HVLT Recognition Discrimination | -0,21 | -2,23 | 0,183 | -0,03 | -0,71 | 1,000 |
| Semantic Fluency (SFT) | -0,09 | -0,8 | 1,000 | -0,04 | -1,14 | 1,000 |
| Letter Number Sequencing (LNS) | 0,04 | 0,39 | 1,000 | -0,05 | -1,32 | 1,000 |
| Symbol Digit Modalities (SDM) | -0,12 | -1,19 | 1,000 | -0,03 | -0,81 | 1,000 |
| **Psychiatric symptoms** |  |  |  |  |  |  |
| Geriatric Depression Scale (GDS) | 0,23 | 3,71 | **<0.001** | 0,02 | 1,56 | 0,240 |
| State-Trait Anxiety Index (STAI) | 0,49 | 5,98 | **<0.001** | 0,01 | 0,52 | 0,604 |

β coefficients with T and p values are derived from linear mixed effect models; p-values are adjusted for FDR. Significant p-values are reported in bold. Abbreviations: UPDRS: Unified Parkinson's disease rating scale; MoCA: Montreal Cognitive Assessment; HVLT: Hopkins verbal learning test.

**Table S6 |** Difference in clinical outcomes between clinical stages PD phenotypes, including as adjunctive covariates cardiovascular disease factor H&Y PD staging and Clonazepam usage.

| **Clinical stages body first vs. brain first phenotype** | **Baseline** | | | **Longitudinal** | | | |
| --- | --- | --- | --- | --- | --- | --- | --- |
|  | β | T value | Adj P | β*time | T value | Adj P |  |
| **Daily life burden** |  |  |  |  |  |  |  |
| UPDRS I | 0.69 | 15.05 | **<0.001** | 0.01 | 1.34 | 0.180 |  |
| UPDRS II | 0.42 | 10.33 | **<0.001** | 0.04 | 3.02 | **0.005** |  |
| **Motor impairment** |  |  |  |  |  |  |  |
| UPDRS III | 0.09 | 2.48 | **0.026** | 0.03 | 2.31 | **0.033** |  |
| Bradykinesia | 0.13 | 3.39 | **0.005** | 0.03 | 2.50 | 0.052 |  |
| Rigidity | 0.07 | 1.47 | 0.282 | 0.01 | 1.00 | 0.634 |  |
| Tremor | -0.05 | -0.82 | 0.413 | -0.01 | -0.64 | 0.634 |  |
| Posture and gait impairment | 0.10 | 2.30 | 0.088 | 0.06 | 3.34 | **0.006** |  |
| Axial | 0.13 | 3.14 | **0.011** | 0.04 | 2.88 | **0.021** |  |
| Upper limbs | 0.07 | 1.69 | 0.272 | 0.02 | 1.45 | 0.441 |  |
| Lower limbs | 0.10 | 2.38 | 0.088 | 0.04 | 3.06 | **0.014** |  |
| UPDRS IV | 0.05 | 1.13 | 0.261 | 0.04 | 2.41 | **0.033** |  |
| **Cognitive funtion** |  |  |  |  |  |  |  |
| MoCA | 0.03 | 0.66 | 1.000 | -0.08 | -5.61 | **<0.001** |  |
| Benton Judgment of Line Orientation | -0.05 | -0.80 | 1.000 | -0.01 | -0.67 | 0.818 |  |
| HVLT Total Recall | -0.05 | -0.79 | 1.000 | -0.02 | -1.41 | 0.485 |  |
| HVLT Recognition Discrimination | -0.09 | -1.30 | 1.000 | -0.01 | -0.83 | 0.818 |  |
| Semantic Fluency | 0.03 | 0.50 | 1.000 | -0.02 | -1.55 | 0.485 |  |
| Letter Number Sequencing | -0.04 | -0.57 | 1.000 | -0.02 | -1.75 | 0.404 |  |
| Symbol Digit Modalities | -0.08 | -1.19 | 1.000 | -0.04 | -3.07 | **0.014** |  |
| **Psychiatric symptoms** |  |  |  |  |  |  |  |
| Geriatric Depression Scale | 0.39 | 6.82 | **<0.001** | 0.03 | 2.33 | **0.041** |  |
| State-Trait Anxiety Index | 0.47 | 8.17 | **<0.001** | 0.00 | 0.49 | 0.627 |  |

β coefficients with T and p values are derived from linear mixed effect models; p-values are adjusted for FDR. Significant p-values are reported in bold. Abbreviations: UPDRS: Unified Parkinson's disease rating scale; MoCA: Montreal Cognitive Assessment; HVLT: Hopkins verbal learning test.

**Table S7 |** Difference in clinical outcomes between prodromal stages PD phenotypes, including as adjunctive covariates cardiovascular disease factor and Clonazepam usage.

| **Clinical stages body first vs. brain first phenotype** | **Baseline** | | | **Longitudinal** | | | |
| --- | --- | --- | --- | --- | --- | --- | --- |
|  | β | T value | Adj P | β*time | T value | Adj P |  |
| **Daily life burden** |  |  |  |  |  |  |  |
| UPDRS I | 0.68 | 12.38 | **<0.001** | -0.01 | -1.22 | 0.222 |  |
| UPDRS II | 0.21 | 7.27 | **<0.001** | 0.02 | 5.49 | **<0.001** |  |
| **Motor impairment** |  |  |  |  |  |  |  |
| UPDRS III | 0.09 | 3.83 | **<0.001** | 0.03 | 7.60 | **<0.001** |  |
| Bradykinesia | 0.07 | 3.12 | **0.009** | 0.02 | 6.98 | **<0.001** |  |
| Rigidity | 0.01 | 0.72 | 0.473 | 0.01 | 2.84 | **0.009** |  |
| Tremor | 0.11 | 3.85 | **0.001** | 0.00 | 0.21 | 0.831 |  |
| Posture and gait impairment | 0.05 | 1.96 | 0.151 | 0.01 | 2.96 | **0.009** |  |
| Axial | 0.04 | 1.74 | 0.164 | 0.02 | 5.19 | **<0.001** |  |
| Upper limbs | 0.12 | 4.72 | **<0.001** | 0.02 | 5.17 | **<0.001** |  |
| Lower limbs | 0.06 | 2.36 | 0.074 | 0.02 | 4.15 | **<0.001** |  |
| UPDRS IV | -0.02 | -0.63 | 0.532 | 0.01 | 2.14 | **0.033** |  |
| **Cognitive funtion** |  |  |  |  |  |  |  |
| MoCA | -0.08 | -1.45 | 0.890 | 0.00 | 0.00 | 1.000 |  |
| Benton Judgment of Line Orientation | -0.04 | -0.49 | 1.000 | 0.00 | 0.05 | 1.000 |  |
| HVLT Total Recall | -0.07 | -0.92 | 1.000 | -0.06 | -2.63 | **0.044** |  |
| HVLT Recognition Discrimination | -0.14 | -1.93 | 0.381 | -0.01 | -0.37 | 1.000 |  |
| Semantic Fluency | 0.00 | 0.04 | 1.000 | -0.06 | -2.54 | **0.045** |  |
| Letter Number Sequencing | 0.05 | 0.67 | 1.000 | -0.08 | -3.65 | **0.002** |  |
| Symbol Digit Modalities | -0.10 | -1.23 | 1.000 | -0.07 | -3.84 | **0.001** |  |
| **Psychiatric symptoms** |  |  |  |  |  |  |  |
| Geriatric Depression Scale | 0.30 | 6.12 | **<0.001** | 0.02 | 2.56 | **0.021** |  |
| State-Trait Anxiety Index | 0.45 | 7.41 | **<0.001** | 0.02 | 2.40 | **0.021** |  |

β coefficients with T and p values are derived from linear mixed effect models; p-values are adjusted for FDR. Significant p-values are reported in bold. Abbreviations: UPDRS: Unified Parkinson's disease rating scale; MoCA: Montreal Cognitive Assessment; HVLT: Hopkins verbal learning test.

**Table S8** |Differences in Locus Coeruleus (LC) gradients between PD clinical and prodromal stages body-first and brain-first phenotypes

|  | **Clinical stages** | | | **Prodromal stages** | | |
| --- | --- | --- | --- | --- | --- | --- |
|  | β | *T value* | *Adj P* | β | *T value* | *Adj P* |
| **x** | -0,06 | -2,43 | **0,020** | -0,02 | -1,06 | 0,521 |
| **y** | 0,12 | 3,23 | **0,004** | -0,04 | -1,16 | 0,521 |
| **z** | -0,24 | -3,42 | **0,003** | 0,08 | 3,80 | **0,001** |
| **x^2^** | -0,03 | -1,02 | 0,310 | -0,01 | -0,35 | 0,724 |
| **y^2^** | -0,11 | -2,83 | **0,008** | 0,02 | 0,68 | 0,646 |
| **z^2^** | 0,10 | 3,05 | **0,005** | -0,01 | -0,40 | 0,724 |
| **x^3^** | 0,07 | 3,10 | **0,005** | 0,01 | 1,36 | 0,521 |
| **y^3^** | -0,05 | -2,29 | **0,026** | 0,01 | 0,67 | 0,646 |
| **z^3^** | 0,07 | 3,73 | **0,002** | -0,04 | -4,48 | **<0,001** |

β coefficients with T and p values are derived from linear models; p-values are adjusted for FDR. Significant p-values are reported in bold.

**Table S9** | **Association between PD phenotypes and SNPs**

| **SNP** | **CHR** | **Nearest gene** | **BP** | **A1** | **A2** | **F-Brain** | **F-Body** | **NMISS** | **OR** | **SE** | **L95** | **U95** | **P-adj** |
| --- | --- | --- | --- | --- | --- | --- | --- | --- | --- | --- | --- | --- | --- |
| rs12497850 | 3 | ***IP6K2*** | 48748989 | G | T | 0.303 | 0.367 | 591 | 0.74 | 0.13 | 0.58 | 0.96 | 0.0212 |
| rs12456492 | 18 | ***RIT2*** | 40673380 | G | A | 0.299 | 0.363 | 591 | 0.75 | 0.13 | 0.59 | 0.96 | 0.0274 |
| rs10748818 | 10 | ***GBF1*** | 104015279 | G | A | 0.174 | 0.129 | 591 | 1.39 | 0.16 | 1.01 | 1.90 | 0.0334 |
| rs9261484 | 6 | ***TRIM40*** | 1439135 | T | C | 0.249 | 0.200 | 591 | 1.35 | 0.14 | 1.02 | 1.78 | 0.0450 |
| rs1293298 | 8 | ***CTSB*** | 11712443 | C | A | 0.237 | 0.289 | 591 | 0.77 | 0.14 | 0.59 | 0.99 | 0.0474 |

Abbreviations: SNP: single-nucleotide polymorphism; CHR: chromosome; BP: base pair position; A1: allele 1; A2: allele 2; F-Brain: frequency in brain-first; F-Body: frequency in body-first; OR: odds ratio; SE: standard error; L95: confidence interval lower limit; U95: confidence interval upper limit.

**Table S10** | Difference in clinical outcomes between genetic polymorphism in prodromal PD stages

|  | β**time* | *T value* | *Adj P* | β**time* | *T value* | *Adj P* |
| --- | --- | --- | --- | --- | --- | --- |
| ***TRIM40* C/C** |  | **C/T** |  |  | **T/T** |  |
| **Motor Impairment** |  |  |  |  |  |  |
| UPDRS III | 0.05 | 8.01 | **0.001** | 0.00 | -0.28 | 1.000 |
| **Cognitive function** |  |  |  |  |  |  |
| MoCA | -0.06 | -3.42 | **<0.001** | -0.04 | -1.09 | 0.825 |
| **Psychiatric symptoms** |  |  |  |  |  |  |
| GDS | 0.00 | 0.09 | 0.932 | 0.00 | 0.01 | 1.000 |
| ***IP6K2* G/G** |  | **T/G** |  |  | **T/T** |  |
| **Motor Impairment** |  |  |  |  |  |  |
| UPDRS III | -0.02 | -2.17 | 0.061 | -0.02 | -2.15 | 0.064 |
| **Cognitive function** |  |  |  |  |  |  |
| MoCA | -0,04 | -1,50 | 0.135 | -0.04 | -1.47 | 0.143 |
| **Psychiatric symptoms** |  |  |  |  |  |  |
| GDS | -0,05 | -2,63 | **0.026** | -0.07 | -3.82 | **<0.001** |
| ***RIT2* A/A** | **A/G** | | | **G/G** | | |
| **Motor Impairment** |  |  |  |  |  |  |
| UPDRS III | -0.01 | -1.82 | 0.103 | -0.03 | -3.02 | **0.008** |
| **Cognitive function** |  |  |  |  |  |  |
| MoCA | 0.03 | 2.00 | 0.103 | 0.01 | 0.28 | 1.000 |
| **Psychiatric symptoms** |  |  |  |  |  |  |
| GDS | -0.02 | -2.12 | 0.103 | 0.00 | -0.19 | 1.000 |
| ***CTSB* A/A** |  | **A/C** |  |  | **C/C** |  |
| **Motor Impairment** |  |  |  |  |  |  |
| UPDRS III | -0.02 | -3.85 | **<0.001** | -0.01 | -0.97 | 0,991 |
| **Cognitive function** |  |  |  |  |  |  |
| MoCA | 0.02 | 1.26 | 0.419 | -0.02 | -0.68 | 0.996 |
| **Psychiatric symptoms** |  |  |  |  |  |  |
| GDS | 0.00 | -0.10 | 0.921 | -0.01 | -0.56 | 0.996 |

β coefficients with T and p values are derived from linear mixed effect models; p-values are adjusted for FDR. Significant p-values are reported in bold. Abbreviations: UPDRS: Unified Parkinson's disease rating scale; MoCA: Montreal Cognitive Assessment; GDS: Geriatric Depression Scale.

**Figure S1| Kaplan-Meier curves comparing PD dementia-free survival between body-first and brain-first PD**


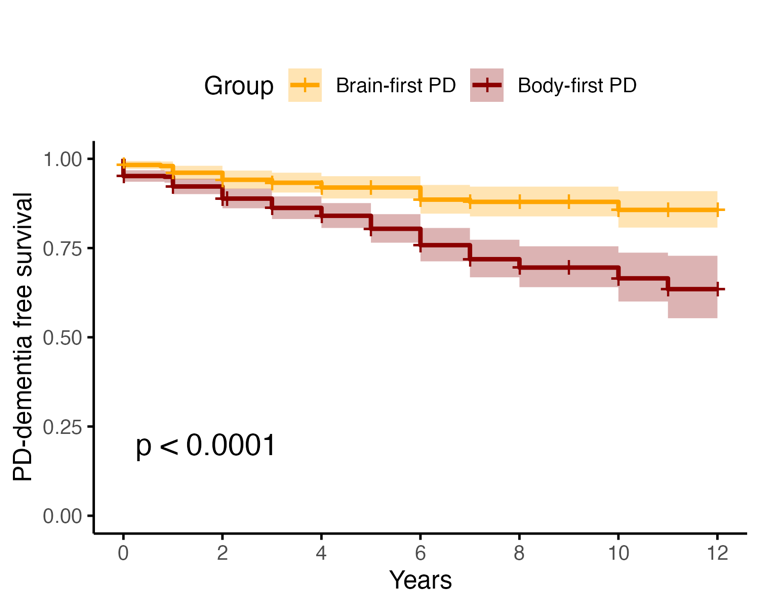
*Kaplan-Meier curves showing PD dementia survival over 12 years in body-first and brain-first PD. Patients in the body-first group exhibited a significantly faster progression to dementia compared to the brain-first group (p < 0.0001). Shaded areas represent 95% confidence intervals.*
